# Dendritic cells direct circadian anti-tumour immune responses

**DOI:** 10.1038/s41586-022-05605-0

**Published:** 2022-12-05

**Authors:** Chen Wang, Coline Barnoud, Mara Cenerenti, Mengzhu Sun, Irene Caffa, Burak Kizil, Ruben Bill, Yuanlong Liu, Robert Pick, Laure Garnier, Olga A. Gkountidi, Louise M. Ince, Stephan Holtkamp, Nadine Fournier, Olivier Michielin, Daniel E. Speiser, Stéphanie Hugues, Alessio Nencioni, Mikaël J. Pittet, Camilla Jandus, Christoph Scheiermann

**Affiliations:** 1 Department of Pathology and Immunology, Faculty of Medicine, University of Geneva, Geneva, Switzerland; 2 Ludwig Institute for Cancer Research, Lausanne, Switzerland; 3 Department of Internal Medicine and Medical Specialties, University of Genoa, Genoa, Italy; 4 AGORA Cancer Research Center, Lausanne, Switzerland; 5 Center for Systems Biology, Massachusetts General Hospital and Harvard Medical School, Boston, MA USA; 6 Department of Computational Biology, University of Lausanne, Lausanne, Switzerland; 7 Swiss Cancer Center Leman, Lausanne, Switzerland; 8 Translational Data Science (TDS), Swiss Institute of Bioinformatics (SIB), Lausanne, Switzerland; 9 Biomedical Center (BMC), Institute for Cardiovascular Physiology and Pathophysiology, Walter-Brendel-Center for Experimental Medicine (WBex), Faculty of Medicine, Ludwig-Maximilians-Universität Munich, Planegg-Martinsried, Germany; 10 Department of Oncology, University of Lausanne, Lausanne, Switzerland; 11 Geneva Centre for Inflammation Research, Geneva, Switzerland; 12 IRCCS Ospedale Policlinico San Martino, Genoa, Italy

**Keywords:** Adaptive immunity, Tumour immunology, Cancer

## Abstract

The process of cancer immunosurveillance is a mechanism of tumour suppression that can protect the host from cancer development throughout its lifetime^1,2^. However, it is unknown whether the effectiveness of cancer immunosurveillance fluctuates over a single day. Here we demonstrate that the initial time of day of tumour engraftment dictates the ensuing tumour size across mouse cancer models. Using immunodeficient mice as well as mice lacking lineage-specific circadian functions, we show that dendritic cells (DCs) and CD8^+^ T cells exert circadian anti-tumour functions that control melanoma volume. Specifically, we find that rhythmic trafficking of DCs to the tumour draining lymph node governs a circadian response of tumour-antigen-specific CD8^+^ T cells that is dependent on the circadian expression of the co-stimulatory molecule CD80. As a consequence, cancer immunotherapy is more effective when synchronized with DC functions, shows circadian outcomes in mice and suggests similar effects in humans. These data demonstrate that the circadian rhythms of anti-tumour immune components are not only critical for controlling tumour size but can also be of therapeutic relevance.

## Abstract (web summary)

Rhythmic trafficking of dendritic cells to the tumour draining lymph node governs a circadian response of tumour-antigen-specific CD8^+^ T cells that is dependent on the circadian expression of the co-stimulatory molecule CD80.

## Main

The immune system provides sophisticated defence mechanisms that most often eliminate or contain the appearance of tumour cells in healthy tissue and prevent the development of life-threatening cancers^1,2^. Both the innate and adaptive arms of immunity show circadian (around 24 h) rhythmicity in their response^3–10^, such that, even weeks after an initial stimulus is encountered, time-of-day immune effects are still observed^11–16^. There is evidence that cancer cells can exhibit a perturbation in their circadian clock components that drives cancer development^17^. However, the effect of a rhythmic immune system on tumour surveillance, and the effectiveness of treatments involving the immune system, remain unclear. Here we provide evidence that a circadian anti-tumour immune response controls tumour volume and the response to therapy.

## Timed engraftment determines tumour size

**Fig. 1:**
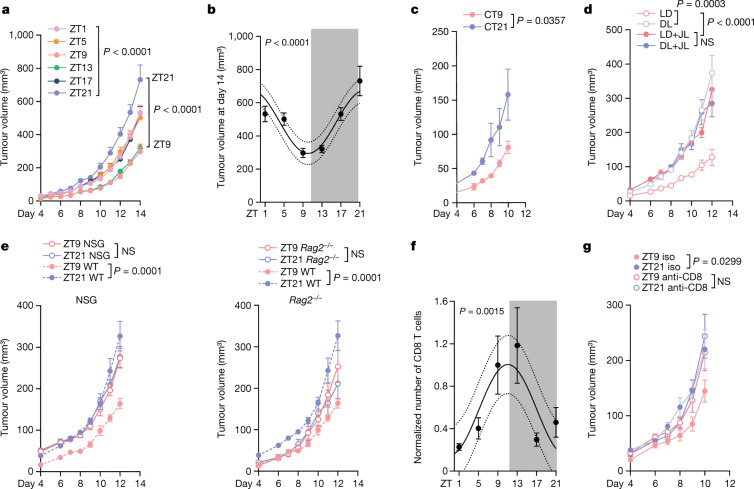
The time of day of engraftment determines tumour size. **a**, Tumour volume after engraftment of B16-F10-OVA cells at six different times of day. *n* = 10 mice from 2 independent experiments. Statistical analysis was performed using two-way analysis of variance (ANOVA). **b**, The tumour volume on day 14 from the experiments shown in **a**. Statistical analysis was performed using a cosinor analysis. **c**, Tumour volume after engraftment of B16-F10-OVA cells at two different times of day under constant darkness (DD) conditions. CT, circadian time. *n* = 6 mice from 2 independent experiments. Statistical analysis was performed using two-way ANOVA. **d**, Tumour volume after engraftment of B16-F10-OVA cells at two different times of day under light–dark (LD, *n* = 6 mice), inverted dark–light (DL, *n* = 7 mice) or jet lag (JL, *n* = 7 mice) conditions. For the jet lag condition, mice were placed into a 6 h or 12 h phase-delayed environment every 3 days. *n* = 2 independent experiments. Statistical analysis was performed using two-way ANOVA. **e**, Tumour volume after engraftment of B16-F10-OVA cells at two different times of day in NSG mice (left, *n* = 10 mice) or *Rag2*^*−/−*^ mice (right). *n* = 10 (ZT9) and *n* = 11 (ZT21) mice. Control WT mice (*n* = 9) are plotted as a reference. Data are from two independent experiments. Statistical analysis was performed using two-way ANOVA. **f**, Tumour-infiltrating CD8^+^ T cells on day 14 from the experiment in **a**. From ZT1 to ZT21, *n* = 10, *n* = 9, *n* = 10, *n* = 7, *n* = 10, *n* = 8 mice from *n* = 4 independent experiments. Statistical analysis was performed using a cosinor analysis. **g**, Tumour volume after engraftment of B16-F10-OVA cells at two different times of day after anti-CD8 antibody depletion. Iso, isotype control. *n* = 6 mice from 2 independent experiments. Statistical analysis was performed using two-way ANOVA. The shaded areas indicate dark phases. For **a**–**g**, data are mean ± s.e.m. NS, not significant. Source data

**Extended Data Fig. 1:**
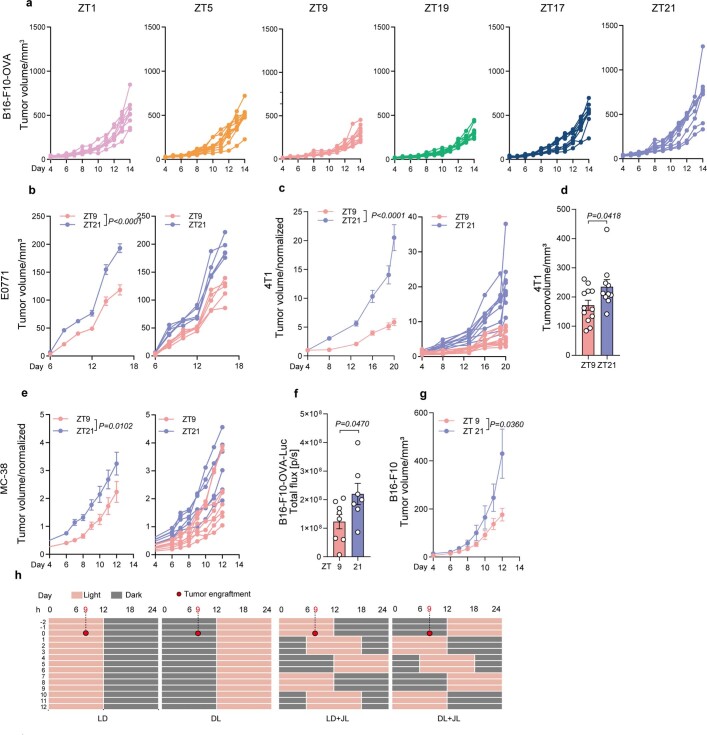
Time of day of engraftment dictates tumour size. (**a**) Tumour volume of each mouse after engraftment of B16-F10-OVA cells at 6 different times of the day (*Zeitgeber* time (ZT)); n = 10 mice per timepoint from 2 independent experiments. (**b**) Tumour volume after engraftment of E0771 cells at 2 different times of the day, plotted per group (left) and mouse (right); n = 5 mice from 2 independent experiments, two-way ANOVA. (**c**-**d**) Normalized tumour volume, plotted per group (left) and mouse (right) (**c**) and tumour volume on day 20 (**d**), after engraftment of 4T1 cells at 2 different times of the day; n = 12 (ZT9), n = 10 (ZT21) mice from 2 independent experiments, two-way ANOVA (**c**) and unpaired Student’s *t*-test (**d**). (**e**) Normalized tumour volume after engraftment of MC-38 cells at 2 different times of the day, plotted per group (left) and mouse (right); n = 8 (ZT9), n = 6 (ZT21) mice from 2 independent experiments, two-way ANOVA. (**f**) Fluorescence flux in photons/seconds of B16-F10-OVA-Luc (5 × 10^5^ cells) tumours on day 5 after engraftment; n = 8 (ZT9), n = 7 (ZT21) mice from 2 independent experiments, unpaired Student’s *t*-test. (**g**) Tumour volume in phase-shifted mice injected using the same batch of B16-F10 cells (without OVA expression); n = 9 (ZT9), n = 6 (ZT21) mice from 2 independent experiments, two-way ANOVA. (**h**) Scheme of the normal (light:dark), inverted lighting (dark:light) and jet lag protocols. For the jet lag, every three days mice were placed into a 6 h- or 12 h-phase delayed environment. The red dots represent the time when tumours were engrafted (9 h after the start of the cycle). All data are represented as mean ± SEM, all *t*-tests are two-tailed. Source data

To examine whether tumour volume depends on the time of day of tumour cell engraftment, we injected B16-F10 melanoma cells expressing ovalbumin (B16-F10-OVA) subcutaneously into cohorts of mice at six different times of day (that is, at zeitgeber time 1 (ZT1; 1 h after light onset in a 12 h–12 h light–dark environment, that is, morning), ZT5 (midday), ZT9 (afternoon), ZT13 (evening), ZT17 (midnight) and ZT21 (early morning)) and quantified tumour size over the ensuing two weeks. To control these data, animals were housed in distinct environmental light cabinets, 12 h phase-shifted to each other, enabling the simultaneous injection of the same batch of tumour cells into differently timed recipients. Tumour size was strongly affected by the time of day of engraftment, yielding significantly larger tumours when inoculated at late night (ZT21), and smaller tumours when inoculated in the late afternoon (ZT9–ZT13) (Fig. 1a,b and Extended Data Fig. 1a). We observed similar results in two orthotopic mammary carcinoma models (E0771 and 4T1) (Extended Data Fig. 1b–d) and a mouse colon carcinoma model (MC-38) (Extended Data Fig. 1e). This indicated that the time-of-day effect of engraftment on tumour size represented a phenomenon that is relevant across different tumour types and sites of engraftment. We further confirmed these data using quantitative imaging approaches with luciferase-expressing melanoma cells (B16-F10-OVA-Luc) (Extended Data Fig. 1f). Experiments using B16-F10 melanoma cell lines that did or did not express OVA yielded very similar results (Extended Data Fig. 1g). This indicated that the observed time-of-day effect of inoculation represented a general phenotype that is not affected by the potentially different immunogenicity of the tumour.

Circadian rhythms are defined by their persistence in the absence of environmental entraining cues, such as rhythmic light onset and offset. Transferring animals to complete darkness conditions did not alter the observed time-of-day-dependent differences, demonstrating that the effect is bona fide circadian in nature (Fig. 1c). However, switching mice to a 12 h inverted dark–light cycle inversed tumour size, demonstrating that the effect was not dependent on light per se but that it could be entrained by light, an additional feature of circadian rhythms (Fig. 1d and Extended Data Fig. 1h). The use of a mouse jet-lag protocol (Extended Data Fig. 1h) abrogated time-of-day differences and increased tumours, indicating that acutely altering lighting regimes negatively affected the disease outcome (Fig. 1d). Together, these data provide evidence that tumour size is highly governed by the initial time of day of engraftment, driven by circadian rhythms in the host that are entrained by light.

## Circadian anti-tumour immune effects

To assess whether these effects were dependent on the immune system, we injected B16-F10-OVA melanoma cells during the day (ZT9) or at night (ZT21) into NSG mice, which lack both adaptive and innate immune cells. Importantly, the previously observed dependency of tumour volume on time-of-day engraftment was abrogated in these mice, indicating that the differences are mediated by the immune system (Fig. 1e). To define which arm of immunity was involved, we used *Rag2*^−/−^ mice, which lack an adaptive immune system. Similar to NSG mice, time-of-day differences in tumour size were ablated in these animals, demonstrating that the adaptive immune system is critical in mediating the phenotype (Fig. 1e).

**Extended Data Fig. 2:**
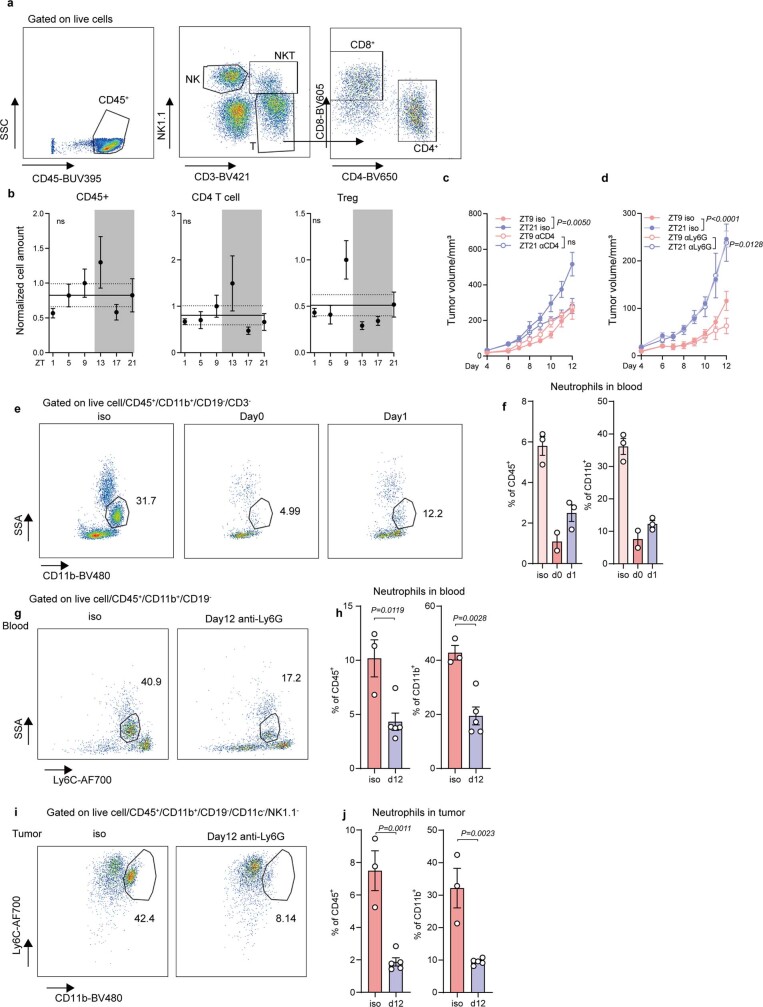
Depletion of CD4 T cells and neutrophils. (**a**) Flow cytometry gating strategy of tumour infiltrating leukocytes. (**b**) Normalized cell numbers of tumour infiltrating leukocytes after 14 days of tumour engraftment; from ZT1 to 21, n = 10, 9, 10, 7, 10, 8 mice from 4 independent experiments, Cosinor analysis. Treg, regulatory T cells. (**c**-**d**) Tumour volume upon anti-CD4 antibody depletion, n = 4 (ZT9 anti-CD4), n = 5 (ZT21 anti-CD4), n = 6 (control) mice (**c**), or anti Ly6G antibody depletion, n = 5 (control), n = 6 (anti-Ly6G) mice (**d**), from 2 independent experiments, two-way ANOVA. (**e**-**j**) Flow cytometry gating strategies and quantification of neutrophils in blood (**e**-**h**) or tumour (**i**-**j**) after anti-Ly6G treatment. Anti-mouse Ly6G antibody was given every 3 days, starting 1 day before the tumour inoculation (d-1). Neutrophil frequency after treatment at days 0 and 1 (**e**-**f**), from left to right, n = 3, 2, 3 mice, or day 12 (**g**-**j**), n = 3 (iso), n = 5 (anti-Ly6G) mice from 2 independent experiments, unpaired Student’s *t*-test. All data are represented as mean ± SEM, ns, not significant, all *t*-tests are two-tailed. Source data

We next used flow cytometry to assess the immune cell infiltrates in tumours 14 days after inoculation. Tumours were collected at ZT1 to limit the variables to only the time of engraftment. The numbers of CD8^+^ T cells were dependent on the time of engraftment, with cellularity peaking when tumour inoculation occurred during the day (ZT9) and troughing at night (ZT21) (Fig. 1f and Extended Data Fig. 2a). By contrast, the numbers of other leukocyte subsets were not affected (Extended Data Fig. 2b). To assess the functional relevance of rhythmicity in the tumour immune cell infiltrate, we used different antibodies to deplete specific subpopulations of leukocytes. Antibody-mediated depletion of CD8^+^ T cells or CD4^+^ T cells—but not of neutrophils—abrogated the time-of-day-dependent differences in tumour size (Fig. 1g and Extended Data Fig. 2c–j). However, only depletion of CD8^+^ T cells increased the tumour volume (Fig. 1g); by contrast, depletion of CD4^+^ T cells decreased the tumour volume (Extended Data Fig. 2c). These results indicate that CD8^+^ T cells exert anti-tumorigenic effects in a time-of-day-dependent manner.

## Rhythmic anti-tumour response in DCs

**Fig. 2:**
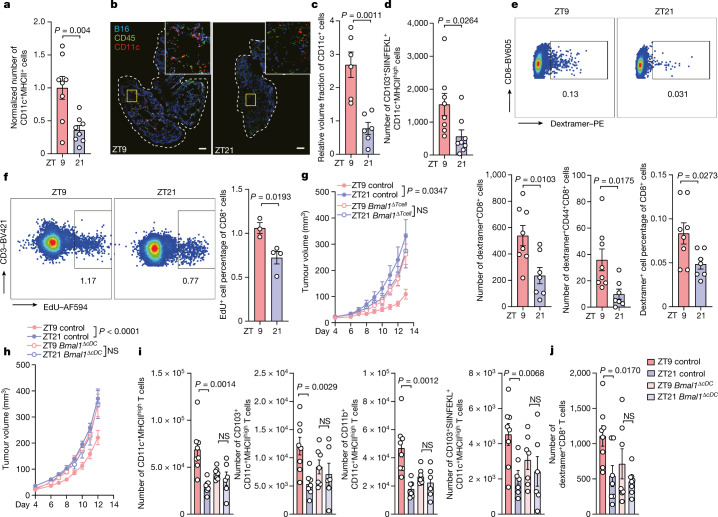
DCs respond rhythmically to tumour engraftment. **a**, The number of cells at the skin engraftment site 4 h after engraftment of B16-F10-OVA cells at two different times of day. *n* = 8 mice from 2 independent experiments. Statistical analysis was performed using unpaired Student’s *t*-tests. **b**,**c**, Imaging (**b**) and quantification (**c**) of CD11c^**+**^ cells at the skin engraftment site 4 h after engraftment of B16-F10-OVA cells. *n* = 6 mice from 2 independent experiments. Statistical analysis was performed using unpaired Student’s *t*-tests. For **b**, scale bars, 500 µm. **d**, The numbers of cells in the dLN 24 h after engraftment of B16-F10-OVA cells. *n* = 8 mice from 2 independent experiments. Statistical analysis was performed using unpaired Student’s *t*-tests. **e**, H-2K^b^–SIINFEKL dextramer staining of CD8^+^ T cells in the dLN 72 h after engraftment of B16-F10-OVA cells. *n* = 8 (ZT9) and *n* = 7 (ZT21) mice from 2 independent experiments. Statistical analysis was performed using unpaired Student’s *t*-tests. **f**, EdU staining gated on CD3^+^CD8^+^ T cells in the dLN 48 h after engraftment of B16-F10-OVA cells. *n* = 3 (ZT9) and *n* = 4 (ZT21) mice, representative of 2 independent experiments. Statistical analysis was performed using unpaired Student’s *t*-tests. **g**,**h**, Tumour volume after engraftment of B16-F10-OVA cells in *Cd4-cre:Bmal1*^*flox*^ mice (*n* = 8 (ZT9 control), *n* = 16 (ZT9 *cre*), *n* = 16 (ZT21 control) and *n* = 7 (ZT21 *cre*)) (**g**) and *Clec9a-cre:Bmal1*^*flox*^ mice (*n* = 17 (ZT9 control), *n* = 9 (ZT9 *cre*), *n* = 16 (ZT21 control) and *n* = 10 (ZT21 *cre*)) (**h**). *n* = 3 independent experiments. Statistical analysis was performed using two-way ANOVA. **i**, The cell numbers for the CD11c^+^MHCII^high^ subsets in the dLN 24 h after engraftment of B16-F10-OVA cells in *Clec9a-cre:Bmal1*^*flox*^ mice. *n* = 8 (ZT9 control), *n* = 7 (ZT9 *cre*), *n* = 7 (ZT21 control) and *n* = 6 (ZT21 *cre*) from 2 independent experiments. Statistical analysis was performed using unpaired Student’s *t*-tests. **j**, H-2K^b^–SIINFEKL dextramer staining of CD8^+^ T cells in the dLN 72 h after engraftment of B16-F10-OVA cells in *Clec9a-cre:Bmal1*^*flox*^ mice. *n* = 8 (ZT9 control), *n* = 7 (ZT9 *cre*), *n* = 7 (ZT21 control) and *n* = 8 (ZT21 *cre*) mice from 2 independent experiments. Statistical analysis was performed using unpaired Student’s *t*-tests. For **a**, **c**, **d** and **f**–**j**, data are mean ± s.e.m. All *t*-tests were two-tailed. Source data

**Extended Data Fig. 3:**
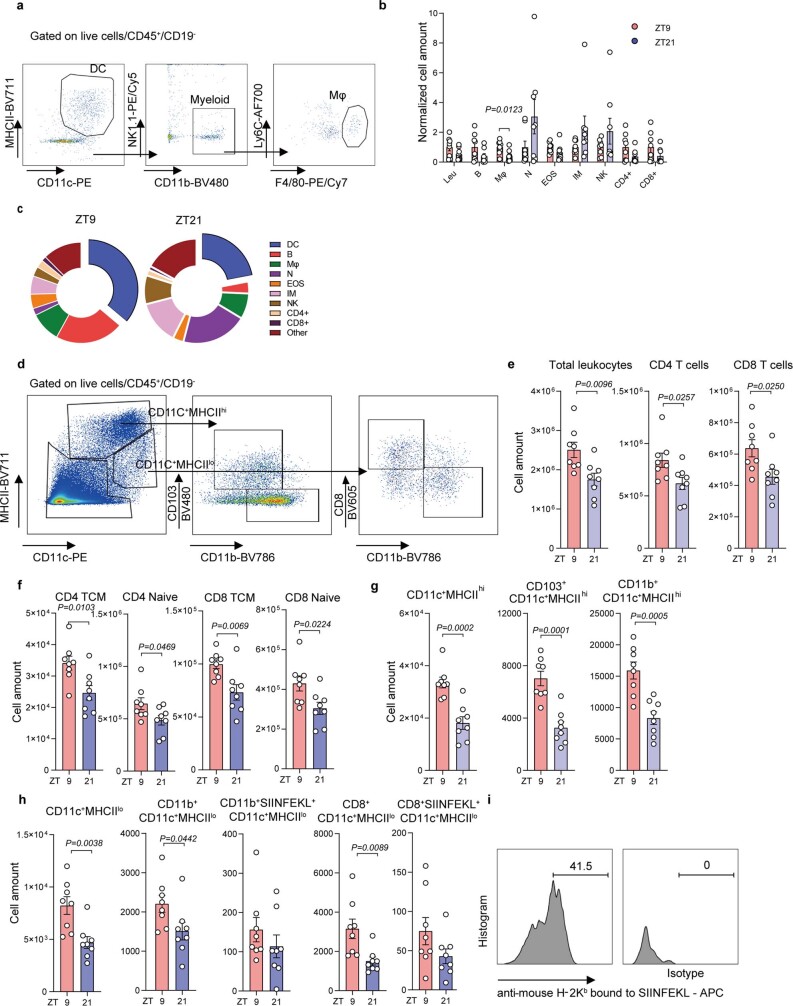
Leukocyte populations in skin and dLN. (**a**) Flow cytometry gating strategy of skin myeloid populations. (**b**-**c**) Number (**b**) and proportion (**c**) of leukocytes at the tumour engraftment site 4 h after B16-F10-OVA engraftment; n = 8 mice from 2 independent experiments, unpaired Student’s *t*-test. Leu, leukocyte; N, neutrophils; EOS, eosinophil; IM, inflammatory monocytes; NK, natural killer cells. (**d**) Flow cytometry gating strategy of DC subsets in draining lymph nodes (dLN). (**e**-**i**) Leukocytes, T cell (**e**-**f**) and DCs (**g**-**h**) in the dLN, 24 h after tumour engraftment (5×10^5^ B16-F10-OVA); n = 8 mice, from 2 independent experiments, unpaired Student’s *t*-test. (**i**) Gating of anti-mouse H-2K^b^ bound to SIINFEKL^+^ DCs in the B16-F10-OVA model. All data are represented as mean ± SEM, all *t*-tests are two-tailed. Source data

**Extended Data Fig. 4:**
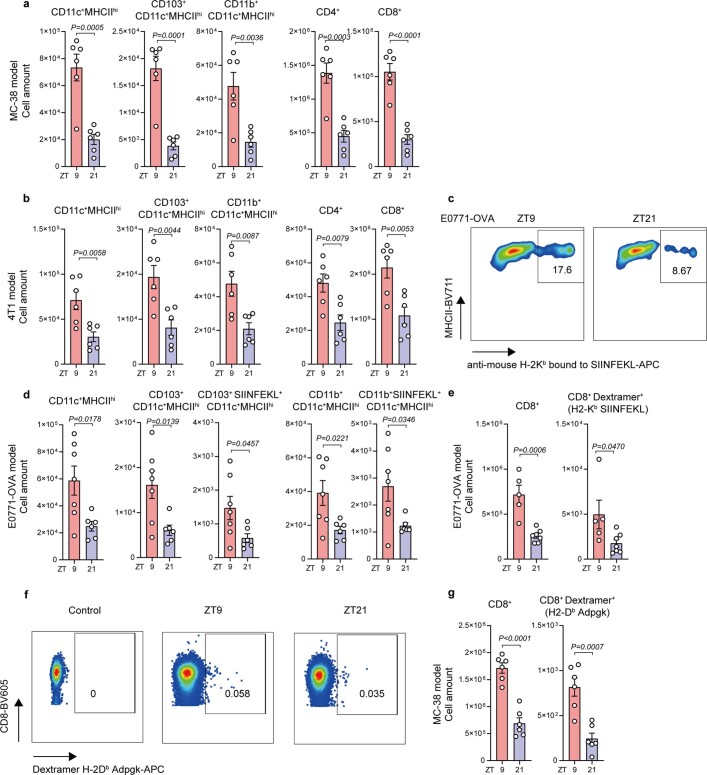
Time-of-day differences exist in other tumour models. (**a**-**d**) DCs and T cell numbers in the dLN, 24h after tumour engraftment of MC-38 cells (n = 6 mice) (**a**), 4T1 cells (n = 6 mice) (**b**), or E0771-OVA cells, n = 7 (ZT9), n = 6 (ZT21) mice, (**c**-**d**), from 2 independent experiments, unpaired Student’s *t*-test. (**c**) Gating of anti-mouse H-2K^b^ bound to SIINFEKL^+^ DCs in the E0771-OVA model. (**e**-**g**) T cell numbers in the dLN 72h after tumour engraftment of E0771-OVA cells, n = 5 (ZT9), n = 7 (ZT21) (**e**), or MC-38 cells, n = 6 mice (**f**-**g**), from 2 independent experiments, unpaired Student’s *t*-test. (**f**) Gating of Dextramer H-2D^b^/ ASMTNMELM bound to CD8 T cells in the MC-38 model and, (**g**) quantification. All data are represented as mean ± SEM, all *t*-tests are two-tailed. Source data

**Extended Data Fig. 5:**
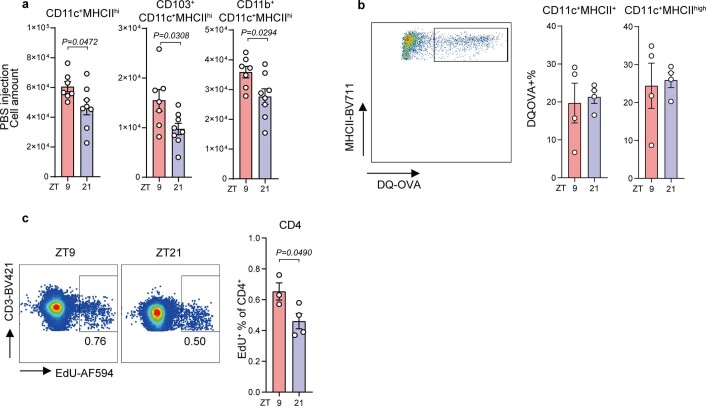
Differences in DCs and CD4 T cells in the dLN. (**a**) DCs numbers in the dLN 24h after sham PBS injection without tumour inoculation, n = 7 (ZT9), n = 8 (ZT21) mice, from 2 independent experiments, unpaired Student’s *t*-test. (**b**) DQ-OVA^+^ DCs in dLN 24h after inoculation; n = 4 mice from 2 independent experiments, unpaired Student’s *t*-test. (**c**) EdU staining in CD3^+^CD4^+^ T cells in the dLN 48 h after B16-F10-OVA cell engraftment; n = 3 (ZT9), n = 4 (ZT21) mice, representative from 2 independent experiments, unpaired Student’s *t*-test. All data are represented as mean ± SEM, all *t*-tests are two-tailed. Source data

To investigate the mechanisms controlling the time-of-day-dependent effect of CD8^+^ T cells on tumour size, we focused on the early events that potentially accounted for the observed effects. Using flow cytometry and quantitative imaging approaches to characterize the site of engraftment 4 h after tumour inoculation, we found that CD11c^+^MHCII^+^ cells represent the predominant leukocyte subset, with higher numbers of these cells when tumour cells were inoculated at ZT9 compared with ZT21 (Fig. 2a–c and Extended Data Fig. 3a–c). Furthermore, 24 h after tumour inoculation, we detected more leukocytes in the draining lymph nodes (dLN) of mice in which tumour cells were inoculated at ZT9 compared with at ZT21 (Extended Data Fig. 3d–e). Specifically, we observed more CD4^+^ and CD8^+^ T cells, including increased numbers of activated central memory (CD44^+^CD62L^+^) and naive (CD44^−^ CD62L^+^) T cells, after tumour cell engraftment at ZT9 (Extended Data Fig. 3e,f). These dLNs also contained more CD11c^+^ cells, including the CD11b^+^CD11c^+^MHCII^high^, CD103^+^CD11c^+^MHCII^high^, CD11b^+^CD11c^+^MHCII^low^ and CD8^+^CD11c^+^MHCII^low^ subsets (Extended Data Fig. 3g–i). This phenotype was also observed in the orthotopic mammary carcinoma as well as the colon carcinoma models (Extended Data Fig. 4a–g). Under sham conditions, time-of-day-dependent changes in the number of these cell types were observed but showed smaller differences (Extended Data Fig. 5a). To further identify relevant tumour-derived antigen-presenting cells (APCs) in the dLN, we used an antibody specific for the SIINFEKL peptide bound to H-2K^b^ (Extended Data Fig. 3h,i). We found that APCs presenting this antigen predominantly displayed a CD103^+^CD11c^+^MHCII^high^ phenotype. These CD103^+^CD11c^+^MHCII^high^(SIINFEKL–H-2K^b^)^+^ cells were also more numerous in the dLNs of mice inoculated with tumour cells at ZT9 (Fig. 2d and Extended Data Fig. 3h,i). This phenotype was also observed in the orthotopic E0771-OVA breast cancer model (Extended Data Fig. 4c,d). No differences were observed in the processing of OVA antigen in CD11c^+^ cells (Extended Data Fig. 5b), suggesting that changes in the levels of antigen presentation were not responsible for the phenotype.

Using dextramer staining to detect endogenous T cells specific for H-2K^b^–SIINFEKL in the dLN 72 h after tumour engraftment, we detected higher numbers and proportions of antigen-specific CD44^+^CD8^+^ T cells when tumour inoculation was performed at ZT9 compared with ZT21 in both the melanoma and mammary carcinoma models (Fig. 2e and Extended Data Fig. 4e). We also observed significantly more EdU^+^CD8^+^ T cells and EdU^+^CD4^+^ T cells in the dLN 48 h after tumour inoculation at ZT9 compared with ZT21, demonstrating a higher T cell proliferation rate after ZT9 tumour inoculation (Fig. 2f and Extended Data Fig. 5c). In an analogous manner, we performed dextramer staining to detect endogenous T cells specific for a peptide of the neoantigen Adpgk (H-2D^b^/ASMTNMELM), expressed by MC-38 colon carcinoma cells. We detected higher numbers of Adpgk^+^-neoantigen-specific CD8^+^ T cells in this tumour model in the dLN 72 h after tumour engraftment when tumour inoculation was performed at ZT9 compared with ZT21 (Extended Data Fig. 4f,g). These data indicate that tumour-antigen-specific CD8^+^ T cells are generated in a time-of-day-dependent manner in the dLN against OVA antigen as well as neoantigen, and similar mechanisms are involved in the subcutaneous and orthotopic engraftment setting.

## Contribution of DC and T cell clocks

To investigate whether the differences were driven by immune-cell-intrinsic mechanisms, we used *Cd4-cre:Bmal1*^*flox*^ mice, in which the key circadian clock gene *Arntl* (also known as *Bmal1*) is specifically deleted in T cells (*Bmal1*^*ΔTcell*^), rendering them arrhythmic. *Bmal1*^*ΔTcell*^ mice showed similar kinetics of tumour volume when tumour cells were inoculated either at ZT9 or ZT21, demonstrating the importance of T-cell-intrinsic rhythms in the control of tumour size (Fig. 2g). Similarly, *Clec9a-cre:Bmal1*^*flox*^ mice, which lack *Bmal1* expression in conventional DCs (*Bmal1*^*ΔcDC*^), showed comparable tumour size kinetics when tumour cells were inoculated either at ZT9 or ZT21 (Fig. 2h). This demonstrated that BMAL1 and cell-autonomous circadian oscillations in both DCs and T cells are critical for the time-of-day differences in tumour volume. Mechanistically, *Bmal1* deletion in cDCs abrogated the differences in total and antigen-specific DC numbers in the dLN after tumour engraftment, in contrast to control mice (Fig. 2i). Furthermore, dextramer staining in *Bmal1*^*ΔcDC*^ mice revealed reduced antigen-specific CD8^+^ T cell levels and abrogated time-of-day-dependent differences (Fig. 2j). These data demonstrate that DC and T cell autonomous circadian clocks are responsible for the time-of-day-dependent anti-tumour effects, with DCs governing rhythmic CD8^+^ T cell responses.

## DCs govern rhythmic anti-tumour immunity

**Fig. 3:**
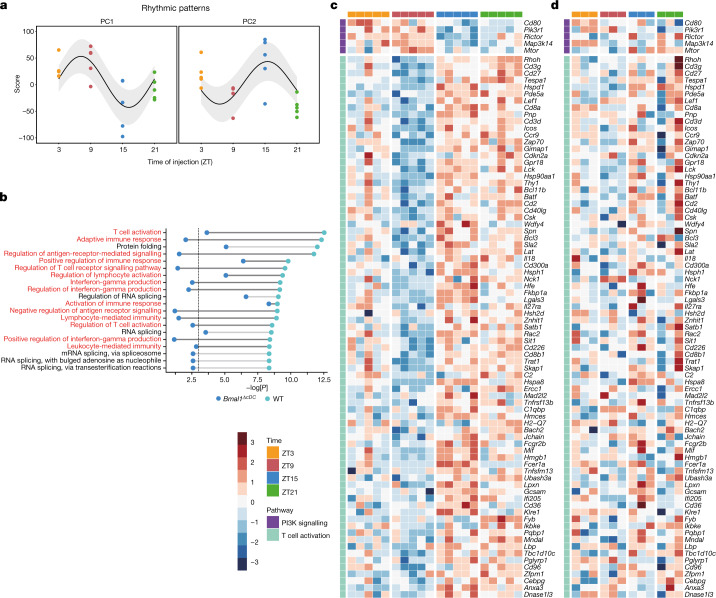
DCs exhibit circadian gene expression patterns. **a**–**d**, RNA-seq analysis of CD11c^+^MHCII^high^ cells in the dLN 24 h after engraftment of B16-F10-OVA cells at ZT3, ZT9, ZT15 or ZT21 in control mice (*n* = 5 mice) or *Clec9a-cre:Bmal1*^*flox*^ mice (*n* = 3 mice). *n* = 2 independent experiments. **a**, Principal component (PC) analyses of the two main peaks in gene expression oscillation in control mice. *n* = 5 mice. Statistical analysis was performed using a cosinor analysis. **b**, Significantly enriched Gene Ontology pathways from PC2 in the control cells shown in **a**, with T cell activation pathways highlighted in red, shown for control and *Clec9a-cre:Bmal1*^*flox*^ CD11c^+^MHCII^high^ cells. The vertical dashed line represents the significant *P* values, which were calculated using hypergeometric tests. **c**,**d**, Significantly expressed genes in the CD28-dependent PI3K–AKT signalling pathway (top) or T cell activation pathways (bottom) in control mice (**c**) and the lack of significance in *Clec9a-cre:Bmal1*^*flox*^ mice (**d**).

**Fig. 4:**
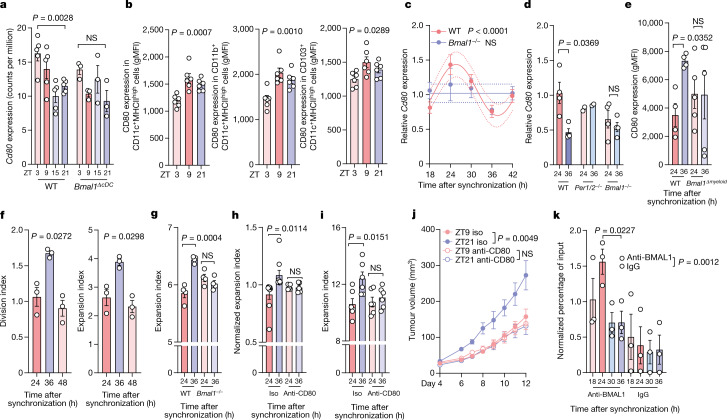
Rhythmic expression of CD80 in DCs governs T cell responses. **a**, Expression (counts per million (CPM)) of *Cd80* in CD11c^+^MHCII^high^ cells from control (*n* = 5) or *Clec9a-cre:Bmal1*^*flox*^ (*n* = 3) mice. Statistical analysis was performed using one-way ANOVA. **b**, CD80 expression in DCs subsets was determined using flow cytometry in the dLN 24 h after engraftment of B16-F10-OVA cells. *n* = 6 mice from 2 independent experiments. Statistical analysis was performed using one-way ANOVA. gMFI, geometric mean fluorescence intensity. **c**, *Cd80* mRNA expression after synchronization of LPS-matured BMDCs from WT (*n* = 10) and *Bmal1*^−/−^ (*n* = 4) mice from 2 independent experiments. Statistical analysis was performed using a cosinor analysis. **d**, *Cd80* mRNA expression after synchronization of BMDCs generated from WT (*n* = 4) *Per1*^*−/−*^*Per2*^*−/−*^ (*n* = 2) or *Bmal1*^−/−^ (*n* = 4) mice. *n* = 2 independent experiments. Statistical analysis was performed using unpaired Student’s *t*-tests. **e**, Floy cytometry analysis of CD80 protein expression in synchronized *Lyz2-cre:Bmal1*^*flox*^ BMDCs. *n* = 4 (control) and *n* = 5 (*cre*) mice from 2 independent experiments. Statistical analysis was performed using paired Student’s *t*-tests. **f**–**h**, In vitro co-culture proliferation experiments with OT-I CD8^+^ T cells and SIINFEKL-loaded BMDCs generated from WT mice (*n* = 3 mice from 2 independent experiments) (**f**) or *Bmal1*^−/−^ mice (*n* = 4, from 2 independent experiments) (**g**) or after anti-CD80 antibody treatment (n = 9 (control) and *n* = 5 (anti-CD80) mice from 9 independent experiments) (**h**). Statistical analysis was performed using one-way ANOVA (**f**), an unpaired Student’s *t*-test (**g**) and a paired Student’s *t*-test (**h**). **i**, In vitro co-culture proliferation experiments with naive CD8^+^ T cells treated with an anti-CD3 antibody and WT BMDCs in the presence of absence of an anti-CD80 antibody. *n* = 3 mice, 2 replicates each, from 2 independent experiments. Statistical analysis was performed using unpaired Student’s *t*-tests. **j**, The tumour volume after engraftment of B16-F10-OVA cells at two different times of day, and after treatment with an anti-CD80 antibody or isotype control. *n* = 10 mice from 2 independent experiments. Statistical analysis was performed using two-way ANOVA. **k**, ChIP analysis of BMAL1 binding to the promoter region of *Cd80* in synchronized BMDCs. *n* = 3 mice from 2 independent experiments. Statistical analysis was performed using unpaired Student’s *t*-tests. For **a**–**k**, all data are mean ± s.e.m. All *t*-tests were two-tailed. Source data

**Extended Data Fig. 6:**
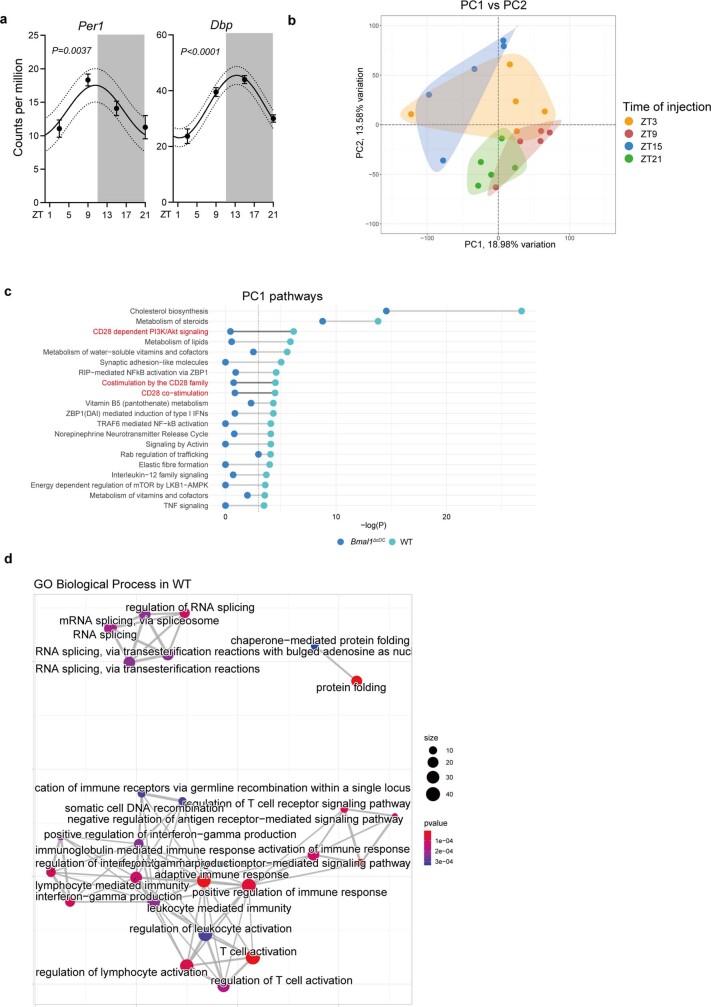
RNA-seq analyses of CD11^+^ MHCII^hi^ cells in the dLN. (**a**-**d**) RNA-seq analyses of CD11c^+^ MHCII^hi^ cells in the dLN 24h after B16-F10-OVA cell engraftment in control mice (n = 5 mice) or *Clec9acre:Bmal1*^*flox*^ mice (n = 3 mice), from 2 independent experiments. (**a**) Expression (Counts per million (CPM)) of *Per1* and *Dbp* in CD11c^+^ MHCII^hi^ cells from control mice, Cosinor analysis. (**b**) Principal Component (PC) analyses of each sample from sequencing of CD11c^+^ MHCII^hi^ DCs in control mice. (**c**) Significantly enriched pathways from PC1 in control cells with CD28 signalling pathways highlighted in red, shown for control and *Clec9acre:Bmal1*^*flox*^ CD11c^+^ MHCII^hi^ cells. The vertical dashed line represents the significant *p* values, hypergeometric test. (**d**) GO Biological Process interactions in the PC2 gene cluster for control cells. All data are represented as mean ± SEM. Source data

**Extended Data Fig. 7:**
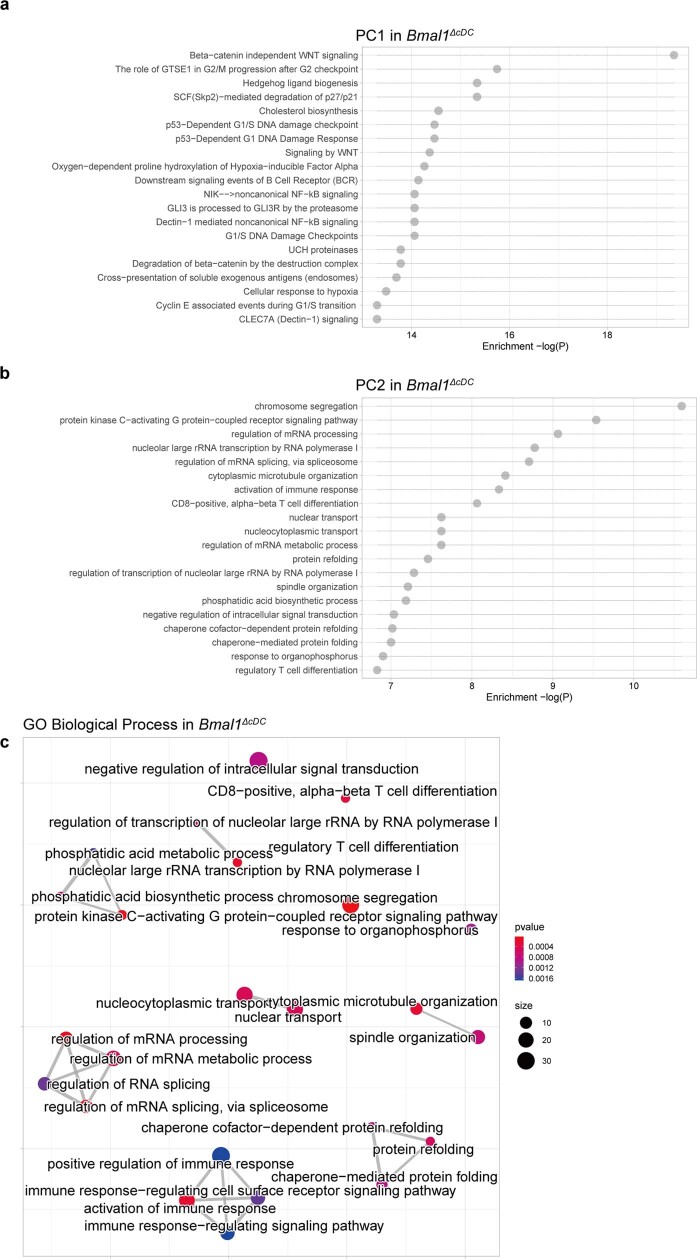
RNA-seq analyses in *Clec9acre:Bmal1*^*flox*^ mice. (**a**-**c**) RNA-seq analyses of CD11c^+^ MHCII^hi^ cells in the dLN 24h after B16-F10-OVA cell engraftment at ZT3, 9, 15 and ZT21 in *Clec9acre:Bmal1*^*flox*^ mice (n = 3 mice). (**a**-**b**) Significantly enriched pathways from PC1 (**a**) and PC2 (**b**) in *Clec9acre:Bmal1*^*flox*^ cells, hypergeometric test. (**c**) GO Biological Process interactions in PC2 gene cluster for *Clec9acre:Bmal1*^*flox*^ cells.

**Extended Data Fig. 8:**
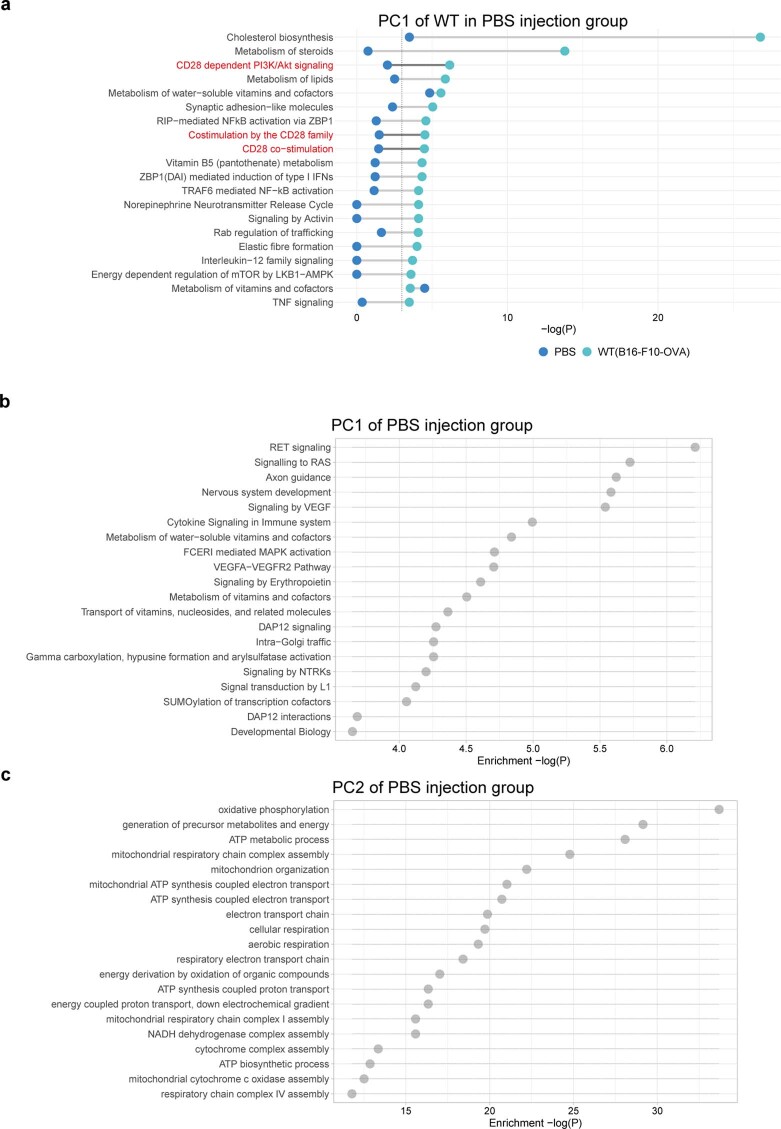
RNA-seq analyses in sham conditions. (**a**-**c**) RNA-seq analyses of CD11c^+^ MHCII^hi^ cells in the dLN 24h after PBS injection (n = 3 mice) or after B16-F10-OVA cell (n = 5 mice) at ZT3, 9, 15 and ZT21 in WT mice, from 2 independent experiments. (**a**) Pathways found significantly enriched by over-representation analysis in the lists of significantly oscillating genes in PC1 (Reactome database) in WT mice. The same pathways from RNA-seq analyses in PBS injection mice were also plotted; n = 3 mice per timepoint. The vertical dashed line represents the significant *P* values, hypergeometric test. (**b**-**c**) Pathways found significantly enriched by over-representation analysis in the lists of significantly oscillating genes in PC1 and PC2 in PBS injection mice, hypergeometric test.

To obtain global information on DC changes after tumour inoculation at different times of the day, we performed RNA-sequencing (RNA-seq) analyses of the subset of CD11c^+^MHCII^high^ migratory DCs in the dLN collected 24 h after tumour engraftment or sham conditions, inoculated at ZT3, ZT9, ZT15 or ZT21. We observed strong time-of-day-dependent differences in overall gene expression, indicating differences in DC functionality. First, we found that CD11c^+^MHCII^high^ cells exhibited rhythmicity in the expression of clock genes and clock-controlled genes (Extended Data Fig. 6a); the expression of these genes was sufficient to define the time of day from which the cells were derived (Extended Data Fig. 6b). Next, we detected two main clusters of oscillatory genes, one of which was expressed more highly in the morning (ZT9) and the other in the evening (ZT15) (Fig. 3a,b). Whereas the morning cluster consisted mainly of metabolic genes—with the exception of the co-stimulatory molecule CD80—the second cluster was highly enriched in T cell activation pathways (Figs. 3b–d and 4a and Extended Data Fig. 6c,d). By contrast, RNA-seq analyses of CD11c^+^MHCII^high^ cells collected from *Bmal1*^*ΔcDC*^ mice showed altered rhythmicity, gene expression patterns and cellular pathways compared with the control mice (Figs. 3b,d and 4a and Extended Data Figs. 6c and  7a–c). These data indicate that, in addition to differences in DC numbers, the rhythmicity in DC co-stimulatory factors that was specific for the tumour scenario (Extended Data Fig. 8a–c) could be responsible for the generation of rhythmic CD8^+^ T cell activation phenotypes.

**Extended Data Fig. 9:**
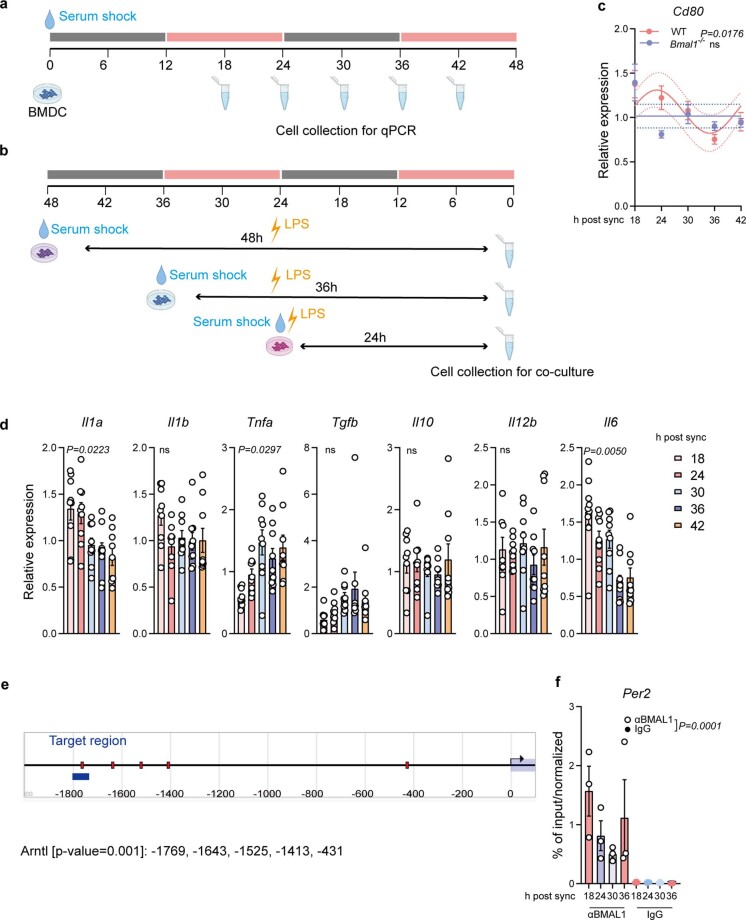
Synchronization experiments of BMDCs. (**a**-**b**) Synchronization scheme of BMDCs for qPCR analyses (**a**) and co-culture experiments (**b**). The schematics in **a** and **b** were created using BioRender. (**c**) *Cd80* mRNA expression after synchronization of immature BMDCs from WT (n = 15 mice) and *Bmal1*^−/−^ (n = 4 mice) mice without LPS maturation, Cosinor analysis. (**d**) qPCR of LPS-matured BMDCs at different times after synchronization; n = 9 mice from 2 independent experiments, one-way ANOVA. (**e**) Predicted binding regions of BMAL1 to the *Cd80* gene using Eukaryotic Promoter Database with a cutoff *P*-value of 0.001. (**f**) Chromatin immunoprecipitation (ChIP) of BMAL1 binding the promoter of *Per2* of BMDCs after synchronization; n = 3 mice, from 2 independent experiments, two-way ANOVA. All data are represented as mean ± SEM, ns, not significant. Source data

CD80 expression was confirmed to be time-of-day dependent in different CD11c^+^ subsets at the protein level on the basis of flow cytometry analysis (Fig. 4b). To investigate whether oscillations in CD80 were driven by a cell-autonomous circadian rhythm—independent of the environment as indicated by the RNA-seq data (Fig. 4a)—we performed in vitro synchronization assays of immature as well as LPS-matured bone-marrow-derived DCs (BMDCs) using a serum shock^18,19^ (Fig. 4c and Extended Data Fig. 9a–c). We detected significant differences in *Cd80* expression at different timepoints after BMDC synchronization (Fig. 4c and Extended Data Fig. 9c). By contrast, we observed no circadian differences in the expression of various cytokines but, rather, a timer-dependent progressive change through time (Extended Data Fig. 9d). Furthermore, circadian differences in *Cd80* expression were abrogated in CD11c^+^MHCII^high^ cells collected from *Bmal1*^*ΔcDC*^ in vivo as well as BMDCs generated from mice lacking overall expression of circadian genes in vitro (*Bmal1*^*−/−*^ or *Per1*^*−/−*^*Per2*^*−/−*^) (Fig. 4a,c,d). To further test whether circadian rhythmicity in myeloid cells was necessary to control *Cd80* expression, we assessed BMDCs produced from mice lacking BMAL1 specifically in myeloid cells (*Lyz2-cre:Bmal1*^*flox*^*;Bmal1*^*Δmyeloid*^); again, we found that the loss of BMAL1 abrogated time-of-day differences in CD80 expression (Fig. 4e). Taken together, these findings indicate a critical role for the circadian clock machinery in controlling CD80 expression.

To investigate whether the circadian rhythmicity in DC gene expression patterns had a functional and causal consequence on T cell activation, we performed co-culture experiments of synchronized, SIINFEKL-pulsed BMDCs with non-synchronized OVA-specific OT-I CD8^+^ T cells. This approach enabled us to observe that the rhythmicity of DCs directly controlled that of T cells. Indeed, the proliferation of OT-I CD8^+^ T cells was highly dependent on the rhythmic phase in which the DCs were located (Fig. 4f). BMDCs collected 36 h after synchronization induced stronger OT-I T cell proliferation compared with BMDCs collected 24 h after synchronization (Fig. 4f). By contrast, BMAL1-deficient BMDCs did not induce rhythmic OT-I CD8^+^ T cell proliferation (Fig. 4g). Furthermore, treatment with an anti-CD80 antibody abrogated time-of-day-dependent differences in OT-I CD8^+^ T cell proliferation (Fig. 4h), demonstrating the relevance of CD80 in the rhythmic CD8^+^ T cell response.

To specifically investigate the importance of rhythmicity in co-stimulatory signals provided by BMDCs, we performed co-culture experiments of synchronized, SIINFEKL-pulsed BMDCs with non-synchronized OVA-specific OT-I CD8^+^ T cells, as before, but bypassing MHCI–TCR interactions using an anti-CD3 antibody. Co-stimulatory signals, in the presence of isotype-matched control antibodies, were sufficient to promote time-of-day-dependent differences in OT-I CD8^+^ T cell proliferation; however, treatment with an anti-CD80 antibody abrogated these time-of-day-dependent differences (Fig. 4i). Finally, antibody treatment against CD80 abrogated the differences in tumour volume after time-of-day-dependent engraftment (Fig. 4j), demonstrating the functional relevance of CD80 in vivo in driving differences in tumour size. These data indicate that CD80 is a critical molecule in this process.

Moreover, we identified the presence of BMAL1-binding sites, namely canonical enhancer boxes (E-boxes), in the promoter region of the *Cd80* gene. This suggests that *Cd80* expression is directly regulated by the circadian clock (Extended Data Fig. 9e). Indeed, using chromatin immunoprecipitation (ChIP) assays, we confirmed rhythmic binding of BMAL1 to the *Cd80* promoter (Fig. 4k and Extended Data Fig. 9f). Together, these data demonstrate that circadian rhythms in DCs direct the rhythms of T cell proliferation, a phenomenon that is dependent on the rhythmic expression of CD80, which is under direct transcriptional control of the clock gene *Bmal1*.

## Vaccination as tumour chrono-immunotherapy

**Fig. 5:**
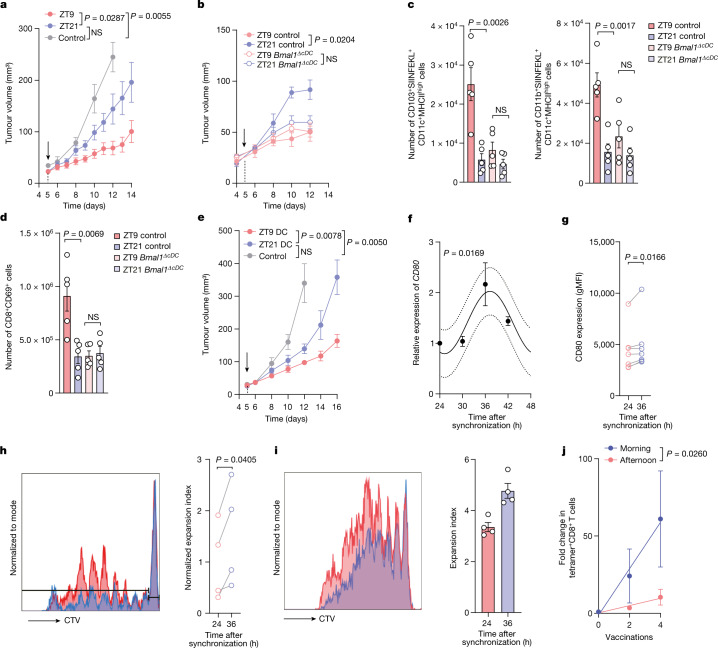
Chronotherapeutic vaccination as tumour immunotherapy. **a**, Tumour volume after engraftment of B16-F10-OVA cells at ZT9 and OVA vaccination on day 5 (arrow) at ZT9 or ZT21. *n* = 12 (vaccinated) and *n* = 3 (unvaccinated control) mice from 2 independent experiments. Statistical analysis was performed using two-way ANOVA. **b**, Tumour volume after engraftment of B16-F10-OVA cells with OVA vaccination on day 5 (arrow) at ZT9 or ZT21 in control or *Clec9a-cre:Bmal1*^*flox*^ mice. *n* = 5 mice from 2 independent experiments. Statistical analysis was performed using two-way ANOVA. **c**,**d**, The cell numbers for DC subsets (**c**) and T cells (**d**) in the dLN 24 h after OVA vaccination (on day 5 after B16-F10-OVA cell engraftment) in control or *Clec9a-cre:Bmal1*^*flox*^ mice. *n* = 5 mice from 2 independent experiments. Statistical analysis was performed using unpaired Student’s *t*-tests. **e**, The tumour volume after engraftment of B16-F10-OVA cells with SIINFEKL-loaded BMDC vaccination on day 5 (arrow) at ZT9 or ZT21. *n* = 6 mice from 2 independent experiments. Statistical analysis was performed using two-way ANOVA. **f**, mRNA expression of *CD80* in hMoDCs after synchronization. *n* = 3 patients. Statistical analysis was performed using a cosinor analysis. **g**, Human CD80 protein expression was analysed using flow cytometry in hMoDCs after synchronization. *n* = 7 patients. Statistical analysis was performed using paired Student’s *t*-tests. **h**, In vitro co-culture proliferation experiments with human naive CD8^+^ T cells and synchronized hMoDCs. *n* = 4 patients. Statistical analysis was performed using paired Student’s *t*-tests. **i**, In vitro co-culture proliferation experiments with antigen-specific CD8^+^ T cells from patients with melanoma and synchronized HLA-A2^+^ hMoDCs. Data are technical replicates, representative of two donors from two independent experiments. Statistical analysis was performed using unpaired Student’s *t*-tests. **j**, Fold change in Melan-A-specific T cells after 2 and 4 vaccinations (with Melan-A peptide, CpG 7909 and incomplete Freund’s adjuvant) in the morning (*n* = 6 patients) or afternoon (*n* = 4 patients). Statistical analysis was performed using a linear regression analysis. For **a**–**g**, **i** and **j**, data are mean ± s.e.m. All *t*-tests were two-tailed. Source data

**Extended Data Fig. 10:**
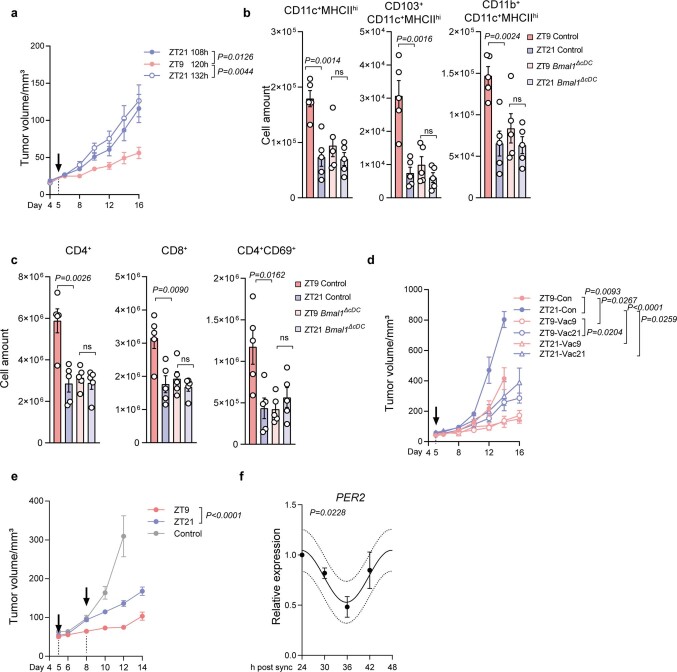
Time-of-day differences in vaccination efficacy. (**a**) Tumour volume after B16-F10-OVA cell engraftment at ZT9 and OVA vaccination at ZT9 (120 h after tumour engraftment, n = 11) or ZT21 (108 h, n = 12, or 132 h, n = 11 mice, after tumour engraftment); from 2 independent experiments, two-way ANOVA. (**b**-**c**) Numbers of DC subsets (**b**) and T cells (**c**) in the draining LN 24h after OVA vaccination (on day 5 after B16-F10-OVA cell engraftment) in control or *Clec9acre:Bmal1*^*flox*^ mice, n = 5 mice from 2 independent experiments, unpaired Student’s *t*-test. (**d**) Tumour volume in WT mice after tumour engraftment (5×10^5^ B16-F10-OVA cells) at ZT9 or ZT21, with or without OVA vaccination on day 5 (arrow) at ZT9 or ZT21, n = 4 mice, two-way ANOVA. (**e**) Tumour volume after B16-F10-OVA cell engraftment at ZT9 and OVA vaccination on day 5 and 8 (arrows), both at ZT9 or ZT21 (n = 6 mice) or unvaccinated controls (n = 9 mice), from 2 independent experiments, two-way ANOVA. (**f**) qPCR of human *PER2* expression in human monocyte derived DCs (hMoDC) after synchronization, n = 3 patients, Cosinor analysis. All data are represented as mean ± SEM, ns, not significant, all *t*-tests are two-tailed. Source data

To evaluate the translational potential of our findings, we examined tumour chrono-immunotherapy. Specifically, we studied mice that were inoculated with B16-F10-OVA melanoma cells at ZT9 and then immunized with OVA either during the day (ZT9) or at night (ZT21). This setting limited the time-of-day information to the timepoint of vaccination only. Notably, we found that tumour volume was strongly suppressed by the vaccine when administered to wild-type (WT) mice at ZT9 compared with ZT21 (Fig. 5a), even when the relative incubation time of the vaccine was substantially longer for ZT21 than for ZT9 (Extended Data Fig. 10a). By contrast, *Bmal1*^*ΔcDC*^ mice vaccinated with OVA at ZT9 or ZT21 showed similar tumour sizes (Fig. 5b). An analysis of dLNs from WT mice 24 h after vaccination revealed a higher number of SIINFEKL-presenting DCs in mice that were vaccinated at ZT9 compared with those vaccinated at ZT21 (Fig. 5c and Extended Data Fig. 10b). Furthermore, this increase at ZT9 coincided with higher numbers of CD69^+^CD8^+^ and CD69^+^CD4^+^ T cells (Fig. 5d and Extended Data Fig. 10c). By contrast, DC and T cell numbers and phenotypes remained similar in *Bmal1*^*ΔcDC*^ mice vaccinated at ZT9 or ZT21 (Fig. 5c,d and Extended Data Fig. 10b,c). These data indicate a key role of cDC rhythmicity in generating a productive anti-tumour immune response after treatment.

We further performed vaccination experiments at ZT9 and ZT21 in a scenario in which tumours were also inoculated at ZT9 and ZT21, therefore assessing the contribution of time-of-day effects in both the timing of tumour inoculation and the timing of vaccination. In these experiments, we observed that the timing of vaccination had a greater impact than the timing of tumour inoculation on tumour burden (Extended Data Fig. 10d). We also confirmed the time-of-day-dependent differences in vaccine efficacy in additional experiments in which two vaccinations were performed at ZT9 or ZT21 several days apart (Extended Data Fig. 10e). These data demonstrate that the timing of vaccination is a powerful means of reducing tumour size, and that rhythmicity of cDCs has a critical role in this process.

To bypass any endogenous DCs acting as potential APCs in this scenario, we performed vaccinations with subcutaneous injections of SIINFEKL-peptide-loaded BMDCs during the day (ZT9) or at night (ZT21). These experiments showed very similar results to the antigen vaccination studies, with suppressed tumour volume after daytime administration of the BMDCs (Fig. 5e). To assess whether these observations could translate to humans, we generated human monocyte-derived DCs (hMoDCs) from CD14^+^ primary monocytes isolated from buffy coats from healthy donors and synchronized them in vitro. hMoDCs exhibited circadian expression of *CD80* as well as of the clock gene *PER2* (Fig. 5f,g and Extended Data Fig. 10f), analogous to the results obtained in mice. Furthermore, co-culture experiments using synchronized hMoDCs together with naive CD8^+^ T cells isolated from the same healthy donors and stimulated with anti-human CD3 antibody—therefore limiting rhythmicity to co-stimulatory factors in DCs only—showed increased T cell proliferation in hMoDCs 36 h after synchronization compared with the 24 h timepoint (Fig. 5h).

Moreover, by generating HLA-A2^+^ MDCs that were pulsed with Melan-A_26–35(A27L)_ peptide (ELAGIGILTV; hereafter, ELA) and co-cultured with HLA-A2/ELA-specific CD8^+^ T cell clones derived from patients with malignant melanoma^20^, we observed time-of-day-dependent differences in the T-cell-proliferation capacity (Fig. 5i). This indicated that the rhythmic anti-tumour responses that we observed in mice were also present in human cells. Indeed, using retrospective time-of-day analyses of a tumour vaccination trial including 10 HLA-A2^+^ patients with advanced malignant melanoma^20^, we observed that time-of-day-dependent differences in vaccine administration result in increased Melan-A-specific CD8^+^ T cells in the patients’ blood, when vaccinations were performed in the morning compared with during the afternoon (Fig. 5j). Together, these data provide evidence for a role of time of day in tumour engraftment and in the efficacy of cancer immunotherapy in mice and humans.

Here we focused mostly on a mouse model of melanoma, while our additional data indicate that other cancer types are also affected by a rhythmic immune system; however, whether similar immune mechanisms are involved in other tumour models remains to be formally demonstrated. Furthermore, our initial patient data indicate the importance of considering the time of day for the administration of cancer immunotherapy. By extension, it is possible that the time of day for the administration of any other treatment that involves activation of the immune system may matter. Given the relative simplicity of controlling this timing parameter in the clinic, it seems important to conduct prospective clinical trials that include sufficient numbers of patients and that can test whether the timing of injection of a given treatment improves the anti-tumour response and the patient’s clinical outcome.

## Methods

### Animals

C57BL/6N and NSG mice were purchased from Charles River. BALB/c mice were purchased from Envigo. *Rag2*^*−/−*^ mice (gift from W. Reith) were bred at Charles River. Other transgenic mouse lines were bred at ENVIGO: *Bmal1*^*flox/flox*^, *Cd4-cre* (both purchased from Jackson Labs) and *Clec9a-cre* (gift from B. Schraml). Transgenic mice were maintained as homozygous for *Bmal1*^*flox/flox*^ and heterozygous for the relevant *cre*. CD45.1 OTI (gift from W. Reith) mice and *Bmal1*^*−/−*^ (gift from C. Dibner) mice were bred in house. All mice used were females at 8–12 weeks of age. Mice were housed under a 12 h–12 h light–dark schedule with food and water ad libitum. When multiple timepoints were investigated simultaneously, light-tight cabinets (Techniplast) were used to shift animals to the respective phase for a minimum of 1 day per 1 h of shift before the experiments. Treatment times correspond to Zeitgeber time (ZT) and indicate the timing relative to lights on in the animal facility such that ZT1 is 1 h after lights on (morning), ZT7 is 7 h after lights on (day time), ZT13 is 1 h after lights off (evening) and ZT19 is 7 h after lights off (night time). Animals were humanely euthanized if the tumour diameter reached 1.5 cm. All of the animal procedures and experiments were approved and performed in accordance with the guidelines of the animal research committee of Geneva, Switzerland or by the Italian Istituto Superiore di Sanità (ISS).

### Tumour cell lines and inoculation

B16-F10 (ATCC) and B16-F10-OVA melanoma cells (from S. Hugues laboratory) were maintained in RPMI (Gibco) supplemented with 10% heat-inactivated FCS (Gibco), 100 μmol l^−1^ penicillin–streptomycin (Gibco) and 50 mmol l^−1^ β-mercaptoethanol (Gibco). B16-F10-OVA-Luc cells were created using ready-to-use lentivirus (GenTarget, LVP324) according to the manufacturer’s instructions. Transduced cells were selected by puromycin (Thermo Fisher Scientific, A1113803) and isolated by fluorescence-activated cell sorting (FACS). MC38 mouse colon adenocarcinoma cells (from S. Hugues laboratory) were maintained in DMEM (Gibco), 10% heat-inactivated FCS, 100 μmol l^−1^ penicillin–streptomycin and 50 mmol l^−1^ β-mercaptoethanol. E0771 and E0771-OVA cells (from S. Hugues laboratory) were maintained in RPMI (Gibco) supplemented with 10% heat-inactivated FCS (Gibco), 100 μmol l^−1^ penicillin–streptomycin (Gibco) and 50 mmol l^−1^ β-mercaptoethanol (Gibco). The 4T1 cell line was purchased from ATCC and maintained in RPMI1640 medium supplemented with 10% heat-inactivated FBS, penicillin (50 U ml^−1^) and streptomycin (50 μg ml^−1^) (LifeTechnologies). Cell lines were used by passage 10 and tested negative for Mycoplasma. Unless otherwise specified, 5 × 10^5^ tumour cells in 100 μl PBS were injected subcutaneously into the right flank of mice, under isoflurane anaesthesia. A total of 5 × 10^5^ 4T1, E0771, or E0771-OVA cells resuspended in PBS were injected orthotopically into the fourth abdominal fat pad of BALB/c (4T1) or C57BL/6 (E0771, E0771-OVA) female mice under ketamine–xylazine anaesthesia. Tumour volume was monitored every 1 to 2 days using callipers and calculated by length × width × width/2. The time of day of measurements did not influence tumour volume (data not shown). In a sham-injection experiment, 100 μl of PBS was injected subcutaneously without tumour cell injection.

### IVIS imaging

d-Luciferin (Abcam, ab143655) was injected intraperitoneally into mice at a dose of 75 mg per kg body weight. Mice were anaesthetized with isoflurane and placed into the abdominal position. Images were collected 8 min after luciferin injection using the IVIS Imaging System (Xenogen), and photons emitted from the tumour were quantified using Living Image Software (Xenogen).

### BMDCs

BMDCs were cultured as previously described^21^, with complete culture medium (RPMI, 10% heat-inactivated FCS, 2 mM l-glutamine, 1% penicillin–streptomycin, 50 µM β-mercaptoethanol) supplemented with 20 ng ml^−1^ recombinant mouse GM-CSF (Peprotech). The medium was refreshed every 3 days. At day 10, all non-adherent and semi-adherent cells were collected in complete medium supplemented with 10 ng ml^−1^ GM-CSF and stimulated with 100 ng ml^−1^ lipopolysaccharide (LPS, L4516, Sigma-Aldrich) for 24 h.

### BMDC synchronization

Cells were synchronized as previously described^18^. In brief, an equal volume of horse serum (Sigma-Aldrich, h1270) was prewarmed and added directly to the dish (serum shock). After incubation for 2 h at 37 °C under 5% CO_2_, cells were washed and resuspended in complete medium.

### Tissue digestion and single-cell preparation

The draining inguinal lymph node was collected and chopped into small pieces, then digested in 1 ml RPMI containing 1 mg ml^−1^ collagenase IV (Worthington Biochemical), 40 μg ml^−1^ DNase I (Roche, 04716728001) and 2% heat-inactivated FCS for 15 min at 37 °C using a thermoblock. Skin tissue was digested in RPMI containing 1 mg ml^−1^ collagenase IV, 2 mg ml^−1^ Dispase II (Roche), 40 μg ml^−1^ DNase I and 2% heat-inactivated FCS for 30 min at 37 °C. Chopped tumour tissue was digested using 1 mg ml^−1^ collagenase IV, 40 μg ml^−1^ DNase I and 2% heat-inactivated FBS for 30 min at 37 °C, and the remaining tumour went through 30 min further digestion using 1 mg ml^−1^ collagenase D (Roche). Cells were rinsed through a 70 μm cell strainer to obtain single-cell suspensions.

### Flow cytometry

Single-cell suspensions were prepared and incubated with mouse or human Fc receptor block (anti-mouse CD16/32, BioLegend, human FcR blocking reagent, Miltenyi Biotec) for 10 min at room temperature. After incubation, unless specified otherwise, the antibody mix was added directly into the cell suspension and incubated for 15 min at 4 °C.

The following anti-mouse antibodies were used for immunostaining: CD45 (30-F11, BUV395, BUV737, BD, 564279, 748371; FITC, BioLegend, 103107), CD45.1 (A20, PE, BioLegend, 110707), CD3e (145-2C11, BUV395, BD563565, APC, BioLegend, 100312; KT3.1.1, BV421, BioLegend, 155617), CD4 (GK1.5, BV650, BD, 563232), CD8a (53-6.7, BV605, BD, 563152; APC, BioLegend, 100711), CD11c (HL3, BUV737, BD612796, N418, PE, BioLegend, 117307), CD19 (1D3, BB700, BD, 566412), CD86 (GL1, BUV395, BD, 564199), CD80 (16-10A1, PE/Cy5, BioLegend, 104711), CD103 (2E7, BV421, BioLegend, 121421), NK1.1 (PK136, PE/Cy5, BioLegend, 108715), MHCII (M5/114.15.2, BV421, BV711, BV650, BioLegend, 107631, 107643, 107641), CD40 (1C10, PerCP-eFluor710, eBioscience, 46-0401-82), CD69 (H1.2F3, BUV737, BD, 612793; BV421, BioLegend, 104527), Ly6G (1A8, BV785, BioLegend, 127645), Ly6C (HK1.4, AF700, BioLegend, 128023). The following anti-human antibodies were used for immunostaining: HLA-DR (G46-6, BV480, BD, 566154), CD11C (B-ly6, BV711, BD, 563130), CD45RA (HI100, PE, BD, 555489), CD25 (2A3, BUV737, BD, 612807), CD44 (G44-26, APC/H7, BD, 560532), CD62L (DREG-56, BV510, BD, 563203), CD8 (RPA-T8, BUV395, BD, 563795), CCR7 (G043H7, BV785, BioLegend, 353230), CD3 (BW264/56, APC, Miltenyi Biotec, 130-113-687).

For peptide–MHC-dextramer staining, 10 µl dextramer (PE-H-2Kb, SIINFEKL, or APC-H-2Db Adpgk, Immudex) were added and incubated at room temperature for 15 min. Anti-mouse H-2K^b^ bound to SIINFEKL antibody staining (25-D1.16, APC, PE/Cy7, BioLegend, 141605) was performed at 37 °C for 15 min. Cells were washed and resuspended in 300 µl FACs buffer with viability dye (DAPI, BioLegend, 3 µM; propidium iodide, Invitrogen, 1.7 µg ml^−1^; or DRAQ7, BioLegend, 2 µM) and characterized using an 18-colour BD LSR Fortessa (BD Biosciences) system or Beckman Coulter Cytoflex. Acquired data were analysed using FACSDiva 6 (BD Biosciences) and FlowJo 10 (BD). Cell counts were calculated using Counting Beads (C36950, C36995, Thermo Fisher Scientific).

When intracellular staining needed to be performed, cells were first stained with viability dye (eBioscience Fixable Viability Dye eFluor 780, 65-0865-18), followed by surface staining as previously described. For intracellular staining, cells were fixed and permeabilized using the FOXP3/Transcription Factor Staining Buffer Set (eBioscience, 00-5523-00). After washing with permeabilization buffer, the intracellular antibody (anti-mouse FOXP3, MF-14, AF647, BioLegend, 126408) was added and incubated for 30 min at room temperature.

### RNA extraction, reverse transcription and qPCR

Cells were collected at the indicated timepoints and lysed using Trizol Reagent (Invitrogen). Tissues were homogenized in Trizol (Invitrogen) using a Precellys 24 (Bertin) bead mill homogenizer. Lysed and homogenized samples were processed using the Direct-zol RNA MiniPrep Kit (Zymo Research) according to the manufacturer’s instructions. RNA quantity and quality was analysed using the Nanodrop 2000 (Thermo Fisher Scientific) or Bioanalyzer. Reverse transcription was performed using the PrimeScript RT Reagent Kit (Takara) according to the manufacturer’s instructions. Quantitative PCR (qPCR) analyses were performed using PowerUp SYBR Green (Applied Biosystems); a list of the primer sequences is provided in Supplementary Table 4. Quantification of the transcript was performed using the 2^−ΔΔ*C*t^ method using *Rplp0*, *Rpl32 *and/or *Gapdh* as internal reference genes.

### In vivo antibody treatments

To deplete specific leukocyte subsets, depletion antibodies or isotype control were injected intraperitoneally 1 day before the tumour inoculation, and repeated every 3 days. The following antibodies were used: anti-mouse CD4 (GK1.5, 100 μg); anti-mouse CD8a (YTS 169.4, 100 μg); anti-mouse Ly6G (1A8, 200 μg), all of which were obtained from BioXCell. For anti-CD80 treatment, 200 μg anti-mouse CD80 antibody (16-10A1, BioXCell) or isotype control (BE0091, BioXCell) were given intraperitoneally 1 day before the tumour inoculation, and repeated every 3 days.

### hMoDCs

Human peripheral blood mononuclear cells (PBMCs) were collected from healthy donors’ buffy coat (provided by the University Hospitals of Geneva (HUG)) using Ficoll-Paque Plus (Cytiva). Monocytes were isolated using the Classical Monocyte Isolation Kit (human, Miltenyi Biotec) according to the manufacturer’s instructions. Cells were centrifuged and resuspended in complete medium (RPMI, 10% heat-inactivated FCS, 2 mM l-glutamine, 1% penicillin–streptomycin, 50 µM β-mercaptoethanol) plus 500 U ml^−1^ human GM-CSF and 250 U ml^−1^ human IL-4 (both from Miltenyi Biotec) and cultured in 12-well cell culture plates at 37 °C with 5% CO_2_. The medium was refreshed every other day. On day 6, non-adherent cells were collected for experiments.

### Proliferation assays

Mouse spleen and lymph nodes were collected from OT-I mice and then passed through a 70 μm cell strainer. After obtaining single-cell suspension, naive CD8^+^ T cells were purified using a cell isolation kit (Miltenyi) according to the manufacturer’s instructions. Cells were adjusted to 2 × 10^6^ cells per ml concentration and stained using the Cell Trace Violet Proliferation Kit (C34557, Invitrogen) at a final concentration of 5 µM in PBS for 15 min at 37 °C. LPS-matured BMDCs were loaded with 10 nM OVA-peptide SIINFEKL for 15 min at 37 °C. Then, 1,000 BMDCs were co-cultured with 10,000 naive CD8^+^ T cells in 200 µl complete medium (RPMI, 10% heat-inactivated FCS, 2 mM l-glutamine, 1% penicillin–streptomycin, 50 µM β-mercaptoethanol, 1 mM pyruvate sodium) in a 96-well round-bottom plate. Plates were incubated at 37 °C with 5% CO_2_ for 48 h before analysis. For polyclonal proliferation assays, naive CD8^+^ T cells were collected from C57BL/6N mice and labelled as described above. A total of 1,000 LPS-matured BMDCs were co-cultured with 10,000 naive CD8^+^ T cells in 200 μl complete medium (RPMI, 10% heat-inactivated FCS, 2 mM l-glutamine, 1% penicillin–streptomycin, 50 µM β-mercaptoethanol, 1 mM pyruvate sodium) supplemented with an anti-mouse CD3 antibody (1 μg ml^−1^, Thermo Fisher Scientific, 16-0032-82) to provide the TCR signal. hMDCs were synchronized using horse serum as described above. Before co-culture with T cells, hMDCs were matured with LPS (200 ng ml^−1^) for 24 h. To assess proliferation, HLA-A2^+^ hMoDCs were used and loaded with Melan-A_26–35(A27L)_ peptide (ELAGIGILTV (ELA), 10 μg ml^−1^)) 24 h before co-culture. For naive T cell proliferation assays, human CD8^+^ T cells were isolated from PBMCs (the same donor as the hMoDCs) using a CD8^+^ T cell isolation kit (Miltenyi Biotec) and then labelled with Cell Trace Violet (Invitrogen). A total of 10,000 labelled T cells were co-cultured with 1,000 matured hMoDCs, together with 5 μg ml^−1^ an anti-human CD3 antibody (Invitrogen, 16-0037-81). Then, 5 days later, T cells were collected for flow cytometry analysis. For patients’ T cell proliferation assays, antigen-specific CD8^+^ T cells were FACS-sorted from PBMCs of patients with melanoma using PE-conjugated HLA-A2/ELA multimers. Multimer^+^ cells were cloned by limiting dilution and expanded with phytohaemagglutinin and allogenic feeder cells in a medium containing 150 U ml^−1^ human recombinant IL-2 (hrIL-2), as previously described^20^. Then, single clones of T cells were used for co-culture with hMoDCs at a ratio of 5:1. On day 5, cells were collected for flow cytometry analysis.

### Vaccinations

Unless specified, 30 μg OVA together with 20 μg CpG OND 1826 and 20 μg poly(I:C) (VacciGrade, all from InvivoGen) were injected subcutaneously into tumour-bearing mice adjacent to the tumour. For vaccination with BMDCs, 1 million SIINFEKL-loaded LPS-matured BMDCs were injected together with 20 μg CpG and 20 μg poly(I:C). The tumour volume was then measured every one or two days using callipers. The human vaccination trial was performed as previously described^20^. The times of vaccination were stratified into vaccinations performed before or after 13:00. The patients included received all of their vaccinations before or after this cut-off time.

### In vitro cell treatment

SIINFEKL-loaded, LPS-matured BMDCs were adjusted to a concentration of 1 × 10^6^ cells per well and incubated with anti-mouse CD80 (50 μg ml^−1^, 16-10A1, BioXCell) or isotype control for 15 min at 37 °C. Proliferation assays were then performed as described above.

### Immunofluorescence imaging

B16-F10-OVA cells were cultured as described above, and labelled with CellTrace Violet (Invitrogen). Cells were counted and resuspended into PBS; an equal volume of cold Corning Matrigel was then added and mixed thoroughly with tumour cells. One million cells in 50 µl were injected subcutaneously into the right flank of the mouse. Then, 4 h later, Matrigels were collected directly into 4% PFA and stored at 4 °C for 4 h. Matrigels were kept in 30% sucrose (Sigma-Aldrich) overnight at 4 °C after fixation, embedded into OCT blocks (CellPath) and kept at −80 °C. Matrigels were subsequently dissected and processed for cryosectioning with 50 µm serial cryosections being cut and processed for immunohistochemistry. The sections were post-fixed with 4% PFA for 10 min at room temperature. After three washes with PBS, the sections were incubated with blocking buffer (PBS with 20% normal goat serum and 0.5% Triton X-100) for 2 h at room temperature. After three consecutive washes with PBS, the sections were stained with an antibody mix of FITC conjugated mouse anti-CD45 (30-F11; BioLegend), PE/Dazzle594-conjugated mouse anti-CD11b (M1/70; BioLegend), Alexa Fluor F647-conjugated mouse anti-CD11c (N418, BioLegend) diluted in the same blocking buffer as before and incubated overnight at 4 °C. The sections were washed three times with PBS before mounting in Fluoromount Aqueous Mounting Medium (Sigma-Aldrich). Images of Matrigels were obtained as sections using the Zeiss Axio Examiner.Z1 confocal spinning-disk microscope equipped with 405-, 488-, 561- and 640-nm laser sources. Step size was determined as 4 µm and images were acquired at ×20 magnification. All image analyses were performed in ImageJ. Volume fractions were obtained from binary images in a 3D environment by thresholding the voxels for both Matrigel volume and the signals of interest.

### Sorting of CD11c^+^MHCII^+^ cells and RNA-seq

To obtain DCs, draining inguinal lymph nodes were collected from mice 24 h after tumour engraftment and collected at four timepoints (ZT3, ZT9, ZT15 and ZT21; *n* = 5 mice for control, *n* = 3 mice for *Clec9acre:Bmal1*^*flox*^ and *n* = 3 mice for sham-injected mice). Lymph nodes were digested as previously described, and CD45^+^CD11c^+^MHCII^high^ cells were sorted using an Astrios sorter (Beckman). Flow-cytometry-sorted DCs were collected in RNAprotect Cell Reagent (76526, Qiagen). RNA was isolated using the RNeasy Plus Micro Kit (74034, Qiagen) according to the manufacturer’s instructions. RNA integrity and quantity were assessed using a Bioanalyzer (Agilent Technologies). cDNA libraries were constructed by the Genomic platform of the University of Geneva as follows: the SMART-Seq v.4 kit from Clontech was used for the reverse transcription and cDNA amplification according to the manufacturer’s specifications, starting with 1 ng of total RNA as the input. Then, 200 pg of cDNA was used for library preparation using the Nextera XT kit from Illumina. Library molarity and quality were assessed using the Qubit and Tapestation using a DNA High sensitivity chip (Agilent Technologies). Libraries were pooled and loaded for clustering on two lanes of a single-read Illumina flow cell. Reads of 50 bases were generated using the TruSeq SBS chemistry on the Illumina HiSeq 4000 sequencer.

Reads were aligned using STAR (v.2.7.0)^22^ to the mouse mm10 UCSC genome. Gene expression was quantified using HTSeq (v.0.9.1)^23^. Differential expression analysis was performed using the R/Bioconductor edgeR package. The counts were normalized according to the library size and filtered. Genes with counts above 1 count per million reads in at least five samples were retained for subsequent analysis. Tests for differentially expressed genes were performed using a general linear model using a negative binomial distribution. The genes were considered to be differentially expressed when the fold change was at least twofold with a 5% false-discovery rate (FDR) Benjamini–Hochberg multiple-testing correction. DiscoRhythm^24^ R package (v.1.10.0) was used to characterize the rhythmicity present in the provided dataset by performing outlier detection, principal component analysis (PCA) and detection of gene-wise oscillation characteristics. The default parameters were used, except when indicated.

PCA was used to extract the strongest recurring patterns in the dataset. Gene expression values were scaled to a s.d. of one before PCA, such that all genes were on an equal scale. The first four PCA scores were used to detect outliers (flagged by their deviation from the mean). A threshold of three units of s.d. was used. The cosinor method was used to test the summarized temporal signal for rhythmicity. PC1 and PC2 were retained as they scored above 10% of the variance each (WT: PC1, 18.9%; PC2, 13.6%; *Bmal1*^*ΔcDC*^: PC1, 20.6%; PC2, 12.3%; PBS sham injection: PC1, 20.2%; PC2, 11.6%) suggesting that two main phases of oscillation exist in the data (Supplementary Tables 1–3). Each gene was tested for rhythmicity with a significance value of *P* < 0.05. Genes with significant rhythmicity were assigned to two sets depending on their acrophase (the time in a periodic cycle during which a temporal pattern is at its maximum value). Genes with a sincoef > 0 corresponded to an acrophase between 0 and 12 h (PC1), whereas genes with a sincoef < 0 corresponded to an acrophase between 12 and 24 h (PC2). Both oscillating gene lists were tested for pathway enrichment, using over-representation analyses in the Gene Ontology Biological Process (GOBP) and Reactome pathways, using ClusterProfiler R^25,26^ package (v.4.4.4). Pathways with an enrichment *P* < 0.05 were reported as significant.

### ChIP with qPCR

A total of 2 × 10^7^ BMDCs were collected and fixed in PBS containing 1% formaldehyde (Thermo Fisher Scientific) for 10 min at room temperature and quenched with 1 M glycine in PBS. Cells were then pelleted and sonicated (Diagenode Bioruptor) to obtain fragments of 0.2–0.8 kilobases in size. Immunoprecipitation was performed using anti-BMAL1 (D2L7G, Cell Signaling Technology), anti-histone H3 (Abcam) or control IgG (Cell Signaling Technology). DNA was isolated using the MinElute PCR Purification kits (Qiagen). qPCR was performed using PowerUp SYBR Green (Applied Biosystems) in the StepOne Real-Time PCR System. Occupancy of BMAL1 at the *Cd80* and *Per2* promoters was quantified by qPCR targeting regions identified as containing E-boxes using the SCOPE motif finder and EPFL eukaryotic database. Relative enrichment was determined as the percentage of input.

### Statistical analyses

Unless otherwise specified, all data were plotted from independent biological replicates. Data were analysed using Prism 9 (GraphPad). *P <  0.05, **P < 0.01, ***P < 0.001, ****P < 0.0001. Unless otherwise specified, Student’s *t*-tests were two-tailed. All other statistical information, including *t*, *F* and d.o.f. values, are provided in the source data.

### Reporting summary

Further information on research design is available in the Nature Portfolio Reporting Summary linked to this article.

## Online content

Any methods, additional references, Nature Portfolio reporting summaries, source data, extended data, supplementary information, acknowledgements, peer review information; details of author contributions and competing interests; and statements of data and code availability are available at 10.1038/s41586-022-05605-0.

## Supplementary information

Supplementary Figures Supplementary Figs. 1–3, showing gating strategies relevant to the Figures and Extended Data Figures.

Reporting Summary

Supplementary Table 1 Full lists of genes significantly (*P* < 0.05) oscillating in CD11c^+^MHCII^high^ cells collected from WT mice in the dLN collected 24 h after subcutaneous B16F10-OVA tumour engraftment in the skin at ZT9 or ZT21 in the PC1 compartment (acrophase < 12), and the PC2 compartment (acrophase > 12). Reactome pathways significantly enriched in PC1 genes, GO biological process pathways in PC2 genes (*P* < 0.05).

Supplementary Table 2 Full lists of genes significantly (*P* < 0.05) oscillating in CD11c^+^MHCII^high^ cells collected from *Bmal1*^*ΔcDC*^ mice in the dLN collected 24 h after subcutaneous B16F10-OVA tumour engraftment in the skin at ZT9 or ZT21 in the PC1 compartment (acrophase < 12), and the PC2 compartment (acrophase > 12). Reactome pathways significantly enriched in PC1 genes, GO biological process pathways in PC2 genes (*P* < 0.05).

Supplementary Table 3 Full lists of genes significantly (*P* < 0.05) oscillating in CD11c^+^MHCII^high^ cells collected from WT mice in the dLN collected 24 h after subcutaneous sham injection (PBS) in the skin at ZT9 or ZT21 in the PC1 compartment (acrophase < 12), and the PC2 compartment (acrophase > 12). Reactome pathways significantly enriched in PC1 genes, GO biological process pathways in PC2 genes (*P* < 0.05).

Supplementary Table 4 Primers used in this study.

## Data Availability

All data supporting the conclusions of this paper are available online (10.26037/yareta:t47xfgrgyvbi3kkg7lfidvzw2q). The sequencing data have been deposited at the NCBI Gene Expression Omnibus (GEO) and are accessible under GEO series accession number GSE217381. Source data are provided with this paper.

## Source data

Source Data Fig. 1
Source Data Fig. 2
Source Data Fig. 4
Source Data Fig. 5
Source Data Extended Data Fig. 1
Source Data Extended Data Fig. 2
Source Data Extended Data Fig. 3
Source Data Extended Data Fig. 4
Source Data Extended Data Fig. 5
Source Data Extended Data Fig. 6
Source Data Extended Data Fig. 9
Source Data Extended Data Fig. 10

## References

[1] Gajewski TF, Schreiber H, Fu YX (2013). Innate and adaptive immune cells in the tumor microenvironment. Nat. Immunol..

[2] Chen DS, Mellman I (2017). Elements of cancer immunity and the cancer-immune set point. Nature.

[3] Arjona A, Silver AC, Walker WE, Fikrig E (2012). Immunity’s fourth dimension: approaching the circadian-immune connection. Trends Immunol.

[4] Curtis AM, Bellet MM, Sassone-Corsi P, O’Neill LA (2014). Circadian clock proteins and immunity. Immunity.

[5] Labrecque N, Cermakian N (2015). Circadian Clocks in the Immune System. J. Biol. Rhythms.

[6] Man K, Loudon A, Chawla A (2016). Immunity around the clock. Science.

[7] Pick, R., He, W., Chen, C. S. & Scheiermann, C. Time-of-day-dependent trafficking and function of leukocyte subsets. *Trends Immunol.* **40**, 524–537 (2019). 10.1016/j.it.2019.03.010 31109762

[8] Scheiermann C, Gibbs J, Ince L, Loudon A (2018). Clocking in to immunity. Nat. Rev. Immunol..

[9] Scheiermann C, Kunisaki Y, Frenette PS (2013). Circadian control of the immune system. Nat. Rev. Immunol..

[10] Palomino-Segura M, Hidalgo A (2021). Circadian immune circuits. J. Exp. Med..

[11] Druzd D (2017). Lymphocyte circadian clocks control lymph node trafficking and adaptive immune responses. Immunity.

[12] Fortier EE (2011). Circadian variation of the response of T cells to antigen. J Immunol.

[13] Silver AC, Arjona A, Walker WE, Fikrig E (2012). The circadian clock controls toll-like receptor 9-mediated innate and adaptive immunity. Immunity.

[14] Sutton CE (2017). Loss of the molecular clock in myeloid cells exacerbates T cell-mediated CNS autoimmune disease. Nat. Commun..

[15] Long JE (2016). Morning vaccination enhances antibody response over afternoon vaccination: a cluster-randomised trial. Vaccine.

[16] de Bree LCJ (2020). Circadian rhythm influences induction of trained immunity by BCG vaccination. J. Clin. Invest..

[17] Papagiannakopoulos, T. et al. Circadian rhythm disruption promotes lung tumorigenesis. *Cell Metab.* **24**, 324–331 (2016). 10.1016/j.cmet.2016.07.001 PMC5367626 27476975

[18] Balsalobre A, Damiola F, Schibler U (1998). A serum shock induces circadian gene expression in mammalian tissue culture cells. Cell.

[19] Holtkamp SJ (2021). Circadian clocks guide dendritic cells into skin lymphatics. Nat. Immunol..

[20] Speiser DE (2005). Rapid and strong human CD8+ T cell responses to vaccination with peptide, IFA, and CpG oligodeoxynucleotide 7909. J. Clin. Invest..

[21] Lutz MB (1999). An advanced culture method for generating large quantities of highly pure dendritic cells from mouse bone marrow. J. Immunol. Methods.

[22] Dobin A (2013). STAR: ultrafast universal RNA-seq aligner. Bioinformatics.

[23] Anders S, Pyl PT, Huber W (2015). HTSeq-a Python framework to work with high-throughput sequencing data. Bioinformatics.

[24] Carlucci M (2019). DiscoRhythm: an easy-to-use web application and R package for discovering rhythmicity. Bioinformatics.

[25] Wu T (2021). clusterProfiler 4.0: A universal enrichment tool for interpreting omics data. Innovation.

[26] Yu G, Wang LG, Han Y, He QY (2012). clusterProfiler: an R package for comparing biological themes among gene clusters. OMICS.

