# Supplementary material for: Dendritic cells direct circadian anti-tumour immune responses
Source: Nature. 2022 Dec 5;614(7946):136–43. doi: 10.1038/s41586-022-05605-0 (PMC9891997; doi:10.1038/s41586-022-05605-0)
Supplement: Supplementary file 1 — Supplementary Figs. 1–3, showing gating strategies relevant to the Figures and Extended Data Figures. [file 41586_2022_5605_MOESM1_ESM.pdf]

nature portfolio

https://doi.org/10.1038/s41586-022-05605-0

Supplementary information

Dendritic cells direct circadian anti-tumour immune responses

In the format provided by the
authors and unedited

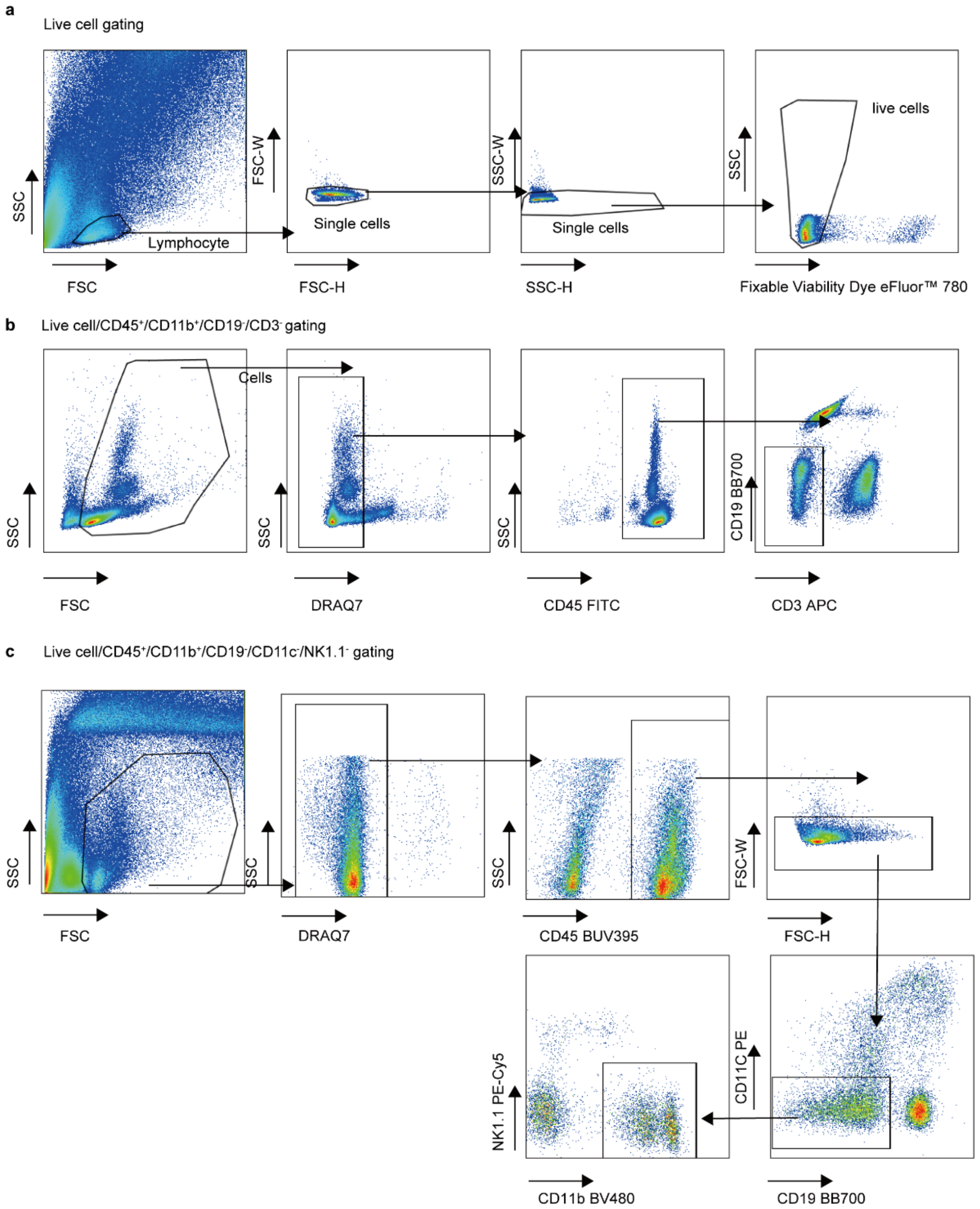

Supplementary Information Figure 1

(a) Gating strategy of CD8 and CD4 T cell in tumor, relevant to Fig 1f, Extended Fig 2b. **(b-c)** Gating strategy relevant to Extended Fig e-i.

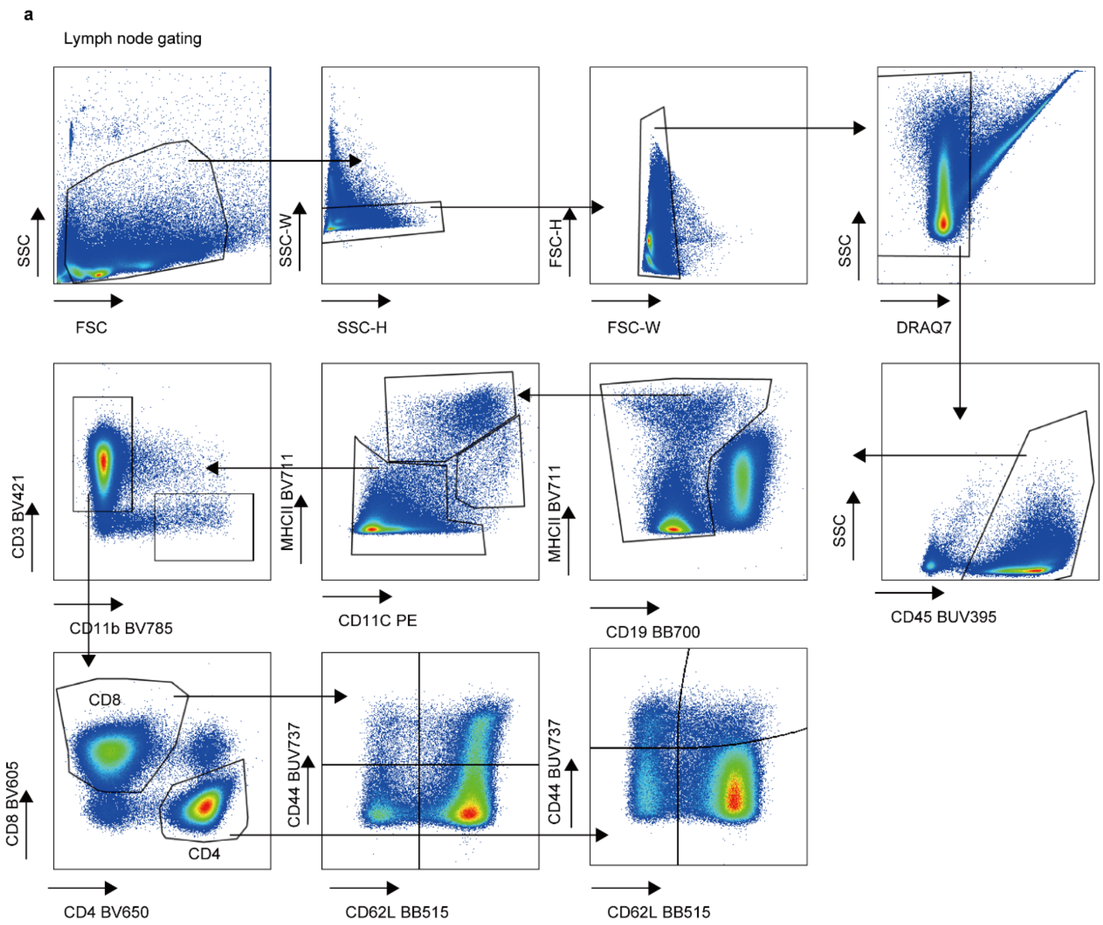

Supplementary Information Figure 2

(a) Gating strategy of cells in lymph node, relevant to Fig 2, 4-5, Extended Fig 3-4,10.

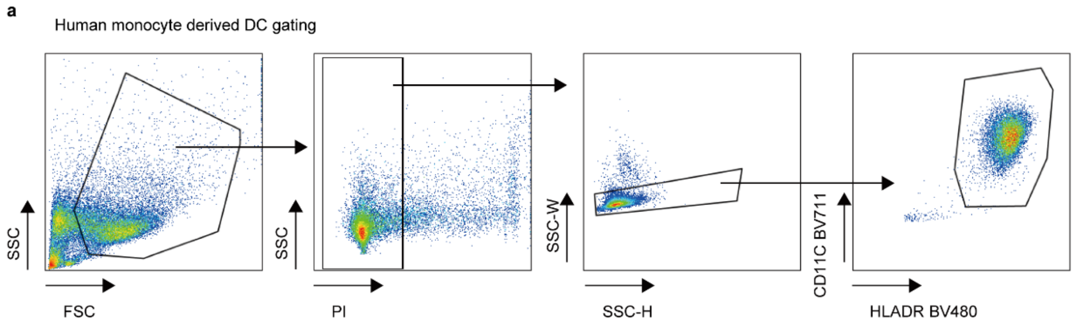

Supplementary Information Figure 3

(a) Gating strategy of human monocyte derived dendritic cell, relevant to Fig 5g.
