# Supplementary material for: Dendritic cells direct circadian anti-tumour immune responses
Source: Nature. 2022 Dec 5;614(7946):136–43. doi: 10.1038/s41586-022-05605-0 (PMC9891997; doi:10.1038/s41586-022-05605-0)
Supplement: Supplementary file 2 — Reporting Summary [file 41586_2022_5605_MOESM2_ESM.pdf]

nature portfolio

Corresponding author(s): Christoph Scheiermann

Last updated by author(s): Nov 9, 2022

# Reporting Summary

Nature Portfolio wishes to improve the reproducibility of the work that we publish. This form provides structure for consistency and transparency in reporting. For further information on Nature Portfolio policies, see our Editorial Policies and the Editorial Policy Checklist.

## Statistics

For all statistical analyses, confirm that the following items are present in the figure legend, table legend, main text, or Methods section.

| n/a | Confirmed | |
|---|---|---|
| ☐ | ☒ | The exact sample size ($n$) for each experimental group/condition, given as a discrete number and unit of measurement |
| ☐ | ☒ | A statement on whether measurements were taken from distinct samples or whether the same sample was measured repeatedly |
| ☐ | ☒ | The statistical test(s) used AND whether they are one- or two-sided<br>*Only common tests should be described solely by name; describe more complex techniques in the Methods section.* |
| ☒ | ☐ | A description of all covariates tested |
| ☐ | ☒ | A description of any assumptions or corrections, such as tests of normality and adjustment for multiple comparisons |
| ☐ | ☒ | A full description of the statistical parameters including central tendency (e.g. means) or other basic estimates (e.g. regression coefficient) AND variation (e.g. standard deviation) or associated estimates of uncertainty (e.g. confidence intervals) |
| ☐ | ☒ | For null hypothesis testing, the test statistic (e.g. $F$, $t$, $r$) with confidence intervals, effect sizes, degrees of freedom and $P$ value noted<br>*Give P values as exact values whenever suitable.* |
| ☒ | ☐ | For Bayesian analysis, information on the choice of priors and Markov chain Monte Carlo settings |
| ☐ | ☒ | For hierarchical and complex designs, identification of the appropriate level for tests and full reporting of outcomes |
| ☒ | ☐ | Estimates of effect sizes (e.g. Cohen's $d$, Pearson's $r$), indicating how they were calculated |

*Our web collection on statistics for biologists contains articles on many of the points above.*

## Software and code

Policy information about availability of computer code

| | |
|---|---|
| Data collection | BD FACSDiva, StepOne, SlideBook, Leica Application Suite, Beckman Coulter Cytoflex |
| Data analysis | Graph Pad Prism v9, ImageJ, FlowJo vl0, R (Discorhythm, R/Bioconductor edgeR, ClusterProfiler V4.4.4, package), Microsoft Excel |

For manuscripts utilizing custom algorithms or software that are central to the research but not yet described in published literature, software must be made available to editors and reviewers. We strongly encourage code deposition in a community repository (e.g. GitHub). See the Nature Portfolio guidelines for submitting code & software for further information.

## Data

Policy information about availability of data

All manuscripts must include a data availability statement. This statement should provide the following information, where applicable:

- Accession codes, unique identifiers, or web links for publicly available datasets
- A description of any restrictions on data availability
- For clinical datasets or third party data, please ensure that the statement adheres to our policy

Bulk sequencing data are available at GSE. All data that support the conclusions of this paper can be found in resource or from the authors on request.

# Field-specific reporting

Please select the one below that is the best fit for your research. If you are not sure, read the appropriate sections before making your selection.

☒ Life sciences ☐ Behavioural & social sciences ☐ Ecological, evolutionary & environmental sciences

For a reference copy of the document with all sections, see nature.com/documents/nr-reporting-summary-flat.pdf

# Life sciences study design

All studies must disclose on these points even when the disclosure is negative.

| | |
|---|---|
| Sample size | For initial experimental design, power analyses were performed to determine sample size. |
| Data exclusions | Data was not excluded, unless suggested via statistical testing (GraphPad, Identitication of outliers, ROUT method, Q=1%). |
| Replication | All experiments were replicated at least once to prove reproducibility and only included if obtained results were the same. |
| Randomization | Mice were randomly allocated into different experimental groups. With respect to human data, due to the retrospective analysis, no randomization was performed. |
| Blinding | Investigators were blind to genotype and/or treatment where applicable. |

# Reporting for specific materials, systems and methods

We require information from authors about some types of materials, experimental systems and methods used in many studies. Here, indicate whether each material, system or method listed is relevant to your study. If you are not sure if a list item applies to your research, read the appropriate section before selecting a response.

## Materials & experimental systems

| n/a | Involved in the study | |
|---|---|---|
| ☐ | ☒ | Antibodies |
| ☒ | ☐ | Eukaryotic cell lines |
| ☒ | ☐ | Palaeontology and archaeology |
| ☐ | ☒ | Animals and other organisms |
| ☐ | ☒ | Human research participants |
| ☒ | ☐ | Clinical data |
| ☒ | ☐ | Dual use research of concern |

## Methods

| n/a | Involved in the study | |
|---|---|---|
| ☒ | ☐ | ChIP-seq |
| ☐ | ☒ | Flow cytometry |
| ☒ | ☐ | MRI-based neuroimaging |

## Antibodies

Antibodies used

The following anti-mouse antibodies were used for immunostaining: CD45 (clone 30-F11, BUV 395, BUV 737, BD,564279,748371, FITC, Biolegend 103107), CD45.1 (A20, PE, Biolegend,110707), CD3e (clone 145-2C11, BUV395,BD563565, APC, Biolegend100312 , clone KT3.1.1, BV421, Biolegend 155617), CD4 (clone GK1.5, BV650, BD 563232), CD8a (clone 53-6.7, BV605, BD 563152, APC, Biolegend 100711), CD11c (clone HL3, BUV737, BD612796, clone N418, PE,Biolegend 117307), CD19 (clone 1D3, BB700,BD 566412), CD86 (clone GL1, BUV395, BD 564199), CD80 (clone 16-10A1, PE/Cy5, Biolegend, 104711), CD103 (clone 2E7, BV421, Biolegend, 121421), NK1.1 (clone PK136, PE/Cy5,Biolegend 108715), MHCII (clone M5/114.15.2, BV421, BV711, BV650,Biolegend 107631,107643,107641), CD40 (clone 1C10, PerCP-eFluor710,eBioscience 46-0401-82), CD69 (clone H1.2F3, BUV737,BD 612793, BV421, Biolegend 104527), Ly6G (clone 1A8, BV785, Biolegend 127645), Ly6C (clone HK1.4, AF700, Biolegend 128023), anti-mouse H-2Kb bound to SIINFEKL antibody (Clone 25-D1.16, APC, PE/Cy7, Biolegend, 141605, 127645), anti-mouse Foxp3 (clone MF-14, AF647,Biolegend 126408).
The following anti-human antibodies were used for immunostaining: HLA-DR (clone G46-6, BV480, BD566154), CD11C (clone B-ly6, BV711, BD563130), CD45RA (clone HI100, PE,BD555489), CD25 (clone 2A3, BUV737, BD612807), CD44 (clone G44-26, APC/H7, BD,560532), CD62L (clone DREG-56, BV510, BD563203), CD8 (clone RPA-T8, BUV395, BD563795), CCR7 (clone G043H7, BV785, Biolegend353230), CD3 (clone BW264/56, APC, Miltenyi Biotec 130-113-687).
For in vivo treatment, anti-mouse CD4, clone GK1.5, 100µg,BE0003-1; anti-mouse CD8a, clone YTS 169.4, 100µg,BE0117; anti-mouse Ly6G, clone 1A8, 200µg, BE0075, all from BioXCell. For anti-CD80 treatment, 200µg anti-mouse CD80 antibody (clone 16-10A1, BioXCell, BE0024) or isotype control (BE0091, BioXCell) were given.
For ChIP, anti-BMAL1 (D2L7G) Rabbit mAb #14020 CST were used.

Validation

Primary antibodies have been validated by the manufacturer for the specific species. All neutralization antibodies used were taken from publications that have validated the antibodies prior to this study.

# Animals and other organisms

Policy information about studies involving animals; ARRIVE guidelines recommended for reporting animal research

| | |
|---|---|
| Laboratory animals | C57BL/6N and NSG mice were purchased from Charles River, BALB/c mice were purchased from Envigo. Rag2-/- mice (gift from Walter Reith, University of Geneva, Switzerland) were bred at Charles River. Other transgenic mouse lines were bred at ENVIGO: Bmal1flox/flox, Cd4cre (both purchased from Jackson Labs) and Clec9acre (gift from Barbara Schraml, LMU Munich, Germany). Transgenic mice were maintained as homozygous for Bmal1flox/flox and heterozygous for the relevant Cre. CD45.1 OTI (gift from Walter Reith) mice and Bmal1-/- (gift from Charna Dibner, University of Geneva, Switzerland) mice were bred in house. All mice used were females at 6-12 weeks of age. |
| Wild animals | The study did not involve wild animals. |
| Field-collected samples | This study did not involve field samples. |
| Ethics oversight | All animal procedures and experiments were approved and performed in accordance with the guidelines of the animal research committee of Geneva, Switzerland, or by the Italian Istituto Superiore di Sanità (ISS). |

Note that full information on the approval of the study protocol must also be provided in the manuscript.

# Human research participants

Policy information about studies involving human research participants

| | |
|---|---|
| Population characteristics | A thorough explanation of all human data is provided in the methods section. PBMCs were from healthy donor`s buffy coat. Antigen specific T cells were from patients with melanoma. More details of the patients` characteristics can be found in Speiser et al JCI 2005. |
| Recruitment | Human buffy coats were collected from blood donors at the University Hospitals of Geneva. Human vaccination data was a retrospectively analysis of a previous publication (Speiser et al JCI 2005). Due to a retrospectively analysis, no additional recruitment was performed. Patients were divided into "morning" or "afternoon" based on the time they received the vaccines. |
| Ethics oversight | Written informed consent was obtained for buffy coats from the healthy donors by the University Hospitals of Geneva. The sampling was conducted according to the Declaration of Helsinki and approved by the Commission Cantonale d'Ethique de la Recherche of the University Hospitals of Geneva. |

Note that full information on the approval of the study protocol must also be provided in the manuscript.

# Flow Cytometry

## Plots

Confirm that:

☒ The axis labels state the marker and fluorochrome used (e.g. CD4-FITC).

☒ The axis scales are clearly visible. Include numbers along axes only for bottom left plot of group (a 'group' is an analysis of identical markers).

☒ All plots are contour plots with outliers or pseudocolor plots.

☒ A numerical value for number of cells or percentage (with statistics) is provided.

## Methodology

| | |
|---|---|
| Sample preparation | Single-cell suspensions were prepared and incubated with mouse or human Fc receptor block (anti-mouse CD16/32 Biolegend, human FcR blocking reagent, Miltenyi Biotec) for 10 minutes at room temperature (RT). After incubation, unless specified otherwise, the antibody mix was added directly into the cell suspension and incubated for 15 min at 4°C.<br>For peptide-MHC-dextramer staining, 10μl dextramer (PE-H-2Kb SIINFEKL, or APC-H-2Db Adpgk, Immudex) were added and incubated at room temperature for 15 min. Anti-mouse H-2Kb bound to SIINFEKL antibody staining (Clone 25-D1.16, APC, PE/Cy7) was performed at 37°C for 15 min. Cells were washed and resuspended in 300 μl FACs buffer with viability dye (DAPI, Biolegend, 3 μM; or Propidium Iodide, Invitrogen, 1.7 μg/ml; or DRAQ7, Biolegend, 2 μM) and characterized using an 18-colour BD LSR Fortessa (BD Biosciences). Acquired data were analyzed using FACSDiva 6 (BD Biosciences) and FlowJo 10 (BD). Cell counts were calculated using Counting Beads (C36950, C36995, ThermoFisher).<br>For intracellular staining, cells were fixed and permeabilized using Foxp3 / Transcription Factor Staining Buffer Set (eBioscience, 00-5523-00). Upon wash with permeabilization buffer, the intracellular antibody (anti-mouse Foxp3, clone MF-14, AF647) was added and incubated for 30 min at room temperature.<br>With respect to FACS, LNs were digested and CD45+CD11C+MHCIIhigh cells were sorted using an Astrios sorter (Beckman). |
| Instrument | 18-colour BD LSR Fortessa (BD Biosciences), Astrios sorter (Beckman). |
| Software | FACSDiva 6 (BD Biosciences) and FlowJo 10 (BD). |

| | |
|---|---|
| Cell population abundance | Post-sort purity was checked after sorting. |
| Gating strategy | After removal of debris, cells were gated by live cells (negative for DAPI or DRAQ7, or PI) and single cells |

☒ Tick this box to confirm that a figure exemplifying the gating strategy is provided in the Supplementary Information.
